# Restricted Versus Liberal Versus Goal-Directed Fluid Therapy for Non-vascular Abdominal Surgery: A Network Meta-Analysis and Systematic Review

**DOI:** 10.7759/cureus.38238

**Published:** 2023-04-28

**Authors:** Timothy Xianyi Yang, Adrian Y Tan, Wesley H Leung, David Chong, Yu Fat Chow

**Affiliations:** 1 Department of Anaesthesiology and Operating Theatre Services, Queen Elizabeth Hospital, Hong Kong, HKG

**Keywords:** intraoperative fluid therapy, systematic literature review, network meta-analysis, perioperative fluid management, general anaesthesia, continuous cardiac output monitoring, goal-directed fluid therapy

## Abstract

Optimal perioperative fluid management is crucial, with over- or under-replacement associated with complications. There are many strategies for fluid therapy, including liberal fluid therapy (LFT), restrictive fluid therapy (RFT) and goal-directed fluid therapy (GDT), without a clear consensus as to which is better.

We aimed to find out which is the more effective fluid therapy option in adult surgical patients undergoing non-vascular abdominal surgery in the perioperative period.

This study is a systematic review and network meta-analysis (NMA) with node-splitting analysis of inconsistency, sensitivity analysis and meta-regression.

We conducted a literature search of Pubmed, Cochrane Library, EMBASE, Google Scholar and Web of Science.

Only studies comparing restrictive, liberal and goal-directed fluid therapy during the perioperative phase in major non-cardiac surgery in adult patients will be included. Trials on paediatric patients, obstetric patients and cardiac surgery were excluded. Trials that focused on goal-directed therapy monitoring with pulmonary artery catheters and venous oxygen saturation (SvO2), as well as those examining purely biochemical and laboratory end points, were excluded.

A total of 102 randomised controlled trials (RCTs) and 78 studies (12,100 patients) were included. NMA concluded that goal-directed fluid therapy utilising FloTrac was the most effective intervention in reducing the length of stay (LOS) (surface under cumulative ranking curve (SUCRA) = 91%, odds ratio (OR) = -2.4, 95% credible intervals (CrI) = -3.9 to -0.85) and wound complications (SUCRA = 86%, OR = 0.41, 95% CrI = 0.24 to 0.69). Goal-directed fluid therapy utilising pulse pressure variation was the most effective in reducing the complication rate (SUCRA = 80%, OR = 0.25, 95% CrI = 0.047 to 1.2), renal complications (SUCRA = 93%, OR = 0.23, 95% CrI = 0.045 to 1.0), respiratory complications (SUCRA = 74%, OR = 0.42, 95% CrI = 0.053 to 3.6) and cardiac complications (SUCRA = 97%, OR = 0.067, 95% CrI = 0.0058 to 0.57). Liberal fluid therapy was the most effective in reducing the mortality rate (SUCRA = 81%, OR = 0.40, 95% CrI = 0.12 to 1.5). Goal-directed therapy utilising oesophageal Doppler was the most effective in reducing anastomotic leak (SUCRA = 79%, OR = 0.45, 95% CrI = 0.12 to 1.5). There was no publication bias, but moderate to substantial heterogeneity was found in all networks.

In preventing different complications, except mortality, goal-directed fluid therapy was consistently more highly ranked and effective than standard (SFT), liberal or restricted fluid therapy. The evidence grade was low quality to very low quality for all the results, except those for wound complications and anastomotic leak.

## Introduction and background

Introduction

Background

Perioperative fluid management is an important factor in perioperative care that contributes to long-term mortality and morbidity [1] and is frequently debated amongst perioperative physicians [2-6]. The three current options are restrictive fluid therapy (RFT), goal-directed fluid therapy (GDT) and liberal fluid therapy (LFT). LFT assists with compensatory intravascular volume expansion, meets physiological requirements and compensates for blood loss, perioperative fasting and redistribution of third space. However, overhydration may lead to tissue oedema with poor wound healing, respiratory and cardiovascular complications and delayed recovery [1]. RFT aims for zero balance, less fluid volume given, with purported reduced perioperative complications [1] and shorter hospital stays. However, it may increase the risk of renal impairment and oliguria. GDT targets various measured hemodynamic variables to optimise hemodynamic status and oxygen delivery. Multiple randomised controlled trials (RCTs) and meta-analyses have been performed comparing the two of these three fluid therapies. Previous meta-analyses concluded that a restrictive approach may be beneficial [2-5]. However, since then, there have been new RCTs on fluid therapies, such as the Restrictive versus Liberal Fluid Therapy for Major Abdominal Surgery (RELIEF) trial in 2018, which concluded that a liberal approach did not increase complication rates and a restrictive approach resulted in increased risks of renal impairment [7]. There is only one updated meta-analysis included up to the RELIEF trial [8], and other later RCTs have still not been analysed in a meta-analysis [8,9].

With the advancement in intraoperative monitoring, novel means of achieving GDT have become available. Zhao et al. [10] recently performed a network meta-analysis (NMA) that compared these different GDTs in patients undergoing all types of surgery but did not compare GDT with other fluid therapies. NMA is an emerging technique for comparing multiple interventions in a single analysis through pooling direct and indirect evidence [11]. It allows different interventions to be compared indirectly if they have also been compared to one common intervention in different RCTs. This study is the first to utilise NMA to compare the many subgroups of GDT with RFT, LFT and standard fluid therapies (SFTs) at the same time.

Objectives

We aimed to investigate which is the more effective fluid therapy strategy in adult surgical patients undergoing non-vascular abdominal surgery during the perioperative period. Is there a difference in the perioperative mortality, length of stay (LOS), complication rates and organ-specific complication rates? This study aims to compare the effectiveness of RFT versus LFT versus GDT in adult patients undergoing non-vascular abdominal surgery during the perioperative period using an NMA and systematic review of the available data.

Methods

Protocol and Registration

We utilised the Preferred Reporting Items for Systematic Reviews and Meta-Analysis Extension Statement for Reporting of Systematic Reviews Incorporating Network Meta-Analyses of Health Care Interventions (PRISMA-NMA) guideline [12]. This work was not registered on any systematic reviews registry.

Inclusion and Exclusion Criteria

Only RCTs comparing the effects of RFT, LFT and GDT during the perioperative phase in major non-cardiac surgery in adult patients were included. Studies of intraoperative and postoperative fluid therapy were included. To support the transitivity assumption in NMA, similar studies were selected [13]. Studies on fluid therapy in cardiothoracic surgery, orthopaedic surgery, neurosurgery and vascular surgery were excluded from the NMA but included in the systematic review. Trials on paediatric patients and obstetric patients were excluded. Only journal articles published in English were included. Articles that did not report the primary or secondary end points were excluded. Trials that focused on GDT with pulmonary artery catheters and venous oxygen saturation (SvO2), as well as those examining purely biochemical and laboratory end points, were excluded. Grey literature such as non-traditional articles including reports, audits, editorials, commentaries, conference reports and abstracts were excluded.

Information Sources and Literature Search

We conducted a literature search of Pubmed, Cochrane Library, EMBASE, Google Scholar and Web of Science. All searches were performed from December 2020 to January 2021. The databases were searched separately to allow mapping to relevant subject headings. The search strategy followed validated methods of the QUOROM statement and Cochrane collaboration [14,15]. We expanded the subject headings search to include all relevant subheadings but restricted to human studies and the English language. Search terms included multiple combinations of Medical Subject Headings (MeSH), such as fluid therapy, surgery, perioperative, operative, complications, restrictive, liberal, goal-directed, FloTrac, esophageal/oesophageal Doppler, pulse pressure variation, optimisation/optimization and others. We did not apply restrictions on publication status or date.

Study Selection and Data Collection Process

Two researchers (YX and AT) independently screened articles by their titles and abstracts to identify eligible studies. Eligible entries then proceeded to full-text review. The references of the identified articles were hand-searched to avoid missing relevant studies. Any new studies found this way had their reference list manually searched. YX and AT used a predesigned data abstraction form to record the trial characteristics, outcomes and information related to the quality of the trial. We scored each trial according to the Jadad scale [16]. Two adjudicators (LL and LW) helped resolve disagreement by consensus. The extracted data was then laid out in a systematic review table (Appendices).

Data Items

The following data were extracted from eligible studies: author name, publication year, study design (whether the randomised controlled trial was single centre or multicentre), surgery type, intervention type and control, intervention phase (preoperative, intraoperative, or postoperative), fluid protocols and hemodynamic monitors and patient number (intervention versus control). The primary outcomes were hospital mortality (both 30-day and 90-day mortality, if reported), length of stay (LOS) (days) and complication rate. When articles reported LOS in terms of median and interquartile range or range, or mean and 95% confidence intervals, the mean and standard deviation were calculated using validated formulas proposed by McGrath et al. [17], Wan et al. [18] and Higgins et al. [19]. The complication rate was reported as the number of patients having at least one complication to avoid double-counting patients and to ensure the event rate was less than the sample size. The secondary outcomes were complication severity (major or minor) and organ-specific complications, which included respiratory complications (respiratory failure, pulmonary oedema, pneumonia and pleural effusion), cardiac complications (cardiac failure, myocardial infarction and arrhythmia), wound infections (both deep and superficial were pooled into a single composite end point), renal complications (including any renal impairment and need for dialysis) and anastomotic leak.

Geometry of the Network

To avoid imbalanced distribution of studies per node and allow for meaningful comparisons between treatment options, we classified the studies based on the authors’ intentions and labels. Following the current literature, we defined RFT, which aimed for zero to negative fluid balance, with minimal physiological maintenance and minimal preloading of intravenous fluid before induction [20]. LFT was defined according to Miller’s Anesthesia [21]. There was no standard definition for SFT amongst different centres, and some studies did not specify it in their protocol. GDT was defined as a protocolised approach using hemodynamic monitors to direct volume replacement to achieve certain hemodynamic goals. We pooled interventions with only one or two studies into a single node (GDTOthers). As the pulse variability index and pulse pressure variation are based on similar concepts, these were pooled to reduce inconsistency (GDTPpv). The majority of GDT studies utilised oesophageal Doppler (GDTOD), Edwards Lifesciences Vigileo/FloTrac (GDTFlo) and lithium dilution cardiac output (LiDCO) (GDTLid). Studies with three arms were included. However, if two arms with the same GDT monitoring modality use different fluids, the data of both arms were pooled.

Risk of Bias Within Individual Studies

We used a modified Cochrane Collaboration Risk of Bias Tool (RoB2) to assess the risk of bias in randomised trials [19]. We assessed selection bias including random sequence generation and allocation concealment, performance bias such as blinding of participants and personnel, detection bias such as blinding of outcome assessment, attrition bias due to incomplete outcome data, and reporting bias due to selective reporting. The overall risk of bias was then assigned to each study and categorised as high risk, low risk or unclear.

Summary Measures

We estimated the comparative efficacy of each fluid therapy using mean difference (MD), odds ratio (OR) and 95% credible intervals (CrI). We then used the surface under cumulative ranking curve (SUCRA) to visualise the treatment hierarchy. For comparisons with significant inconsistency, we report the direct, indirect and network evidence with comments on the reliability of the evidence.

Methods of Statistical Analysis

We used RevMan 5.4 to produce a risk of bias assessment and summary as per the Cochrane guide [19]. For the NMA, we used R version 4.0.3 (R Foundation for Statistical Computing, Vienna, Austria), Rstudio version 1.4.1103, and the gemtc, rjags, dmetar and netmeta packages to perform the statistical analysis [22]. All statistical analysis was performed by researcher YX and corroborated by AT. We selected a Bayesian approach for better coverage and estimation of effects in terms of probabilities [23-25]. We performed Markov chain Monte Carlo (MCMC) simulations using a random effects model, and final estimates were based on stable posterior sampling after initial iterations were discarded. We assessed the simulations for convergence using trace plots and the Brooks-Gelman-Rubin statistic and assessed the density plots for normal distribution. The potential scale reduction factor (PSRF) was below 1.05 for all simulations, indicating reliability. SUCRA scores were given to each intervention using the models.

Assessment of Inconsistency and Additional Analyses

We evaluated the consistency of the network models using the node split method with significant inconsistency defined as P < 0.05. When we identified inconsistency, we reviewed the extracted data to ensure that there were no errors and examined the potential effect modifiers of the studies. We performed further sensitivity analyses to improve the robustness of the results. We used multivariate network meta-regression to analyse the remaining inconsistency. The deviance information criterion (DIC) was assessed, and we took a DIC of more than five to indicate that there were substantial differences in the models with and without the covariate [26]. The Grading of Recommendations Assessment, Development and Evaluation (GRADE) guidelines were used to assess the quality of evidence of each network [27]. Evidence was graded as high quality, moderate quality, low quality or very low quality.

Risk of Bias Across Studies

We assessed heterogeneity for each outcome using I^2^ and defined I^2^ of 75% and greater as substantial heterogeneity, 25%-75% as moderate heterogeneity and below 25% as low heterogeneity as defined by Higgins et al. [28]. We used a comparison-adjusted funnel plot and Egger’s test to evaluate the risk of publication bias [29]. An asymmetrical funnel plot and a P-value of <0.1 on Egger’s test indicated the presence of publication bias.

## Review

Results

Study Selection

**Figure 1:**
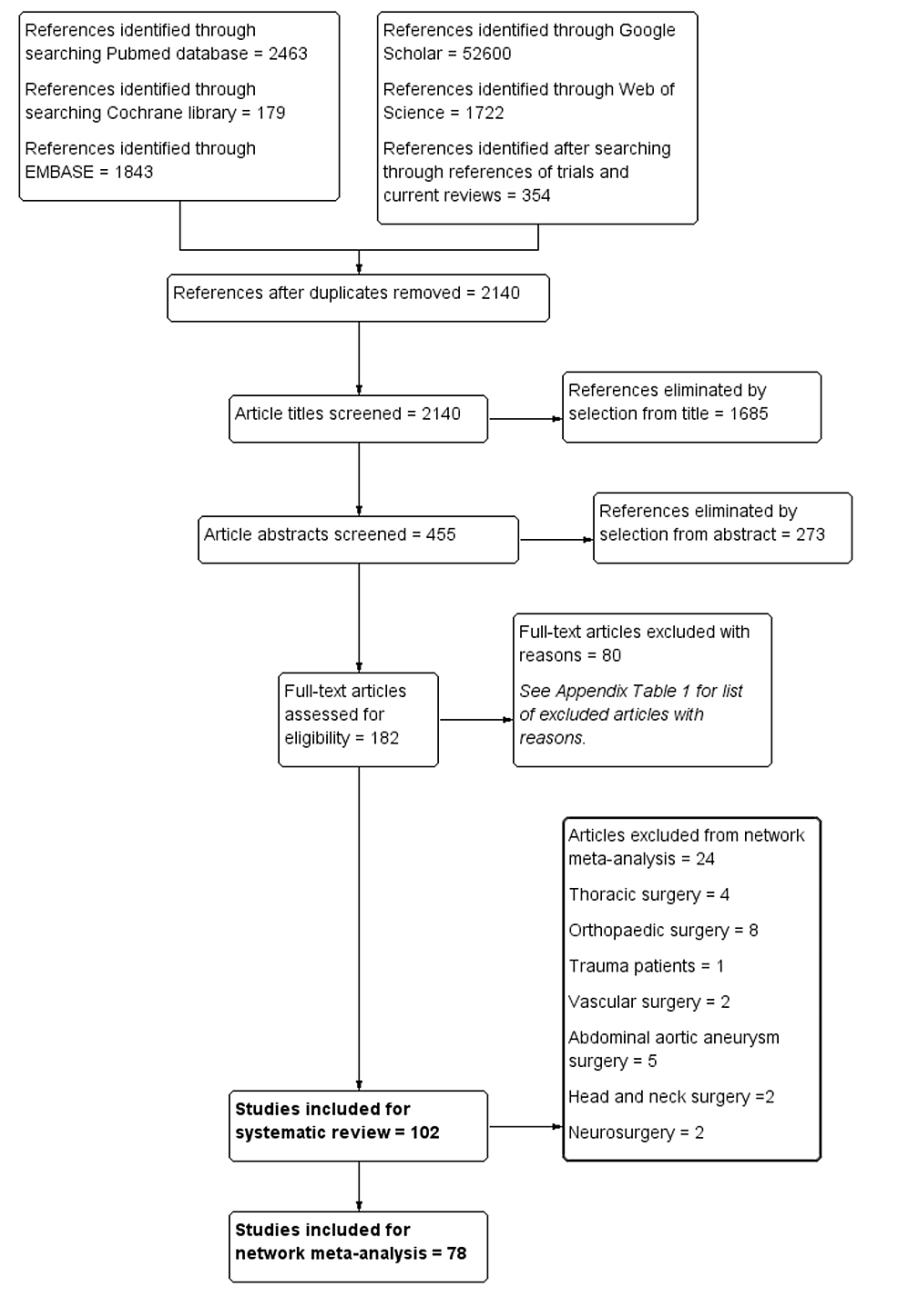
Study selection diagram

After a search and removal of duplicates, we screened 2,140 article titles and eliminated 1,685 studies. We assessed 182 articles for eligibility and included 102 articles in the systematic review [7,30-132], while 78 articles were eligible for network meta-analysis (Figure 1). The excluded studies and their reasons for exclusion, and the included studies and their characteristics can be found in the Appendices.

Study Characteristics

Sixteen studies looked at RFT versus SFT, eight studies compared RFT to GDT, 10 studies compared RFT to LFT and 68 studies compared GDT to SFT. LOS, mortality and complications were reported by 85, 83 and 77 studies, respectively. In terms of secondary outcomes, wound complications, anastomotic leak, renal complications, respiratory complications and cardiac complications were reported by 64, 43, 63, 75 and 71 studies, respectively. Fifty studies were assessed to have a low risk of bias, while 52 studies had an unclear or high risk of bias. In total, there were 14,017 patients included in the systematic review, with a median number of 80 patients per study.

Summary of Network Geometry

**Figure 2:**
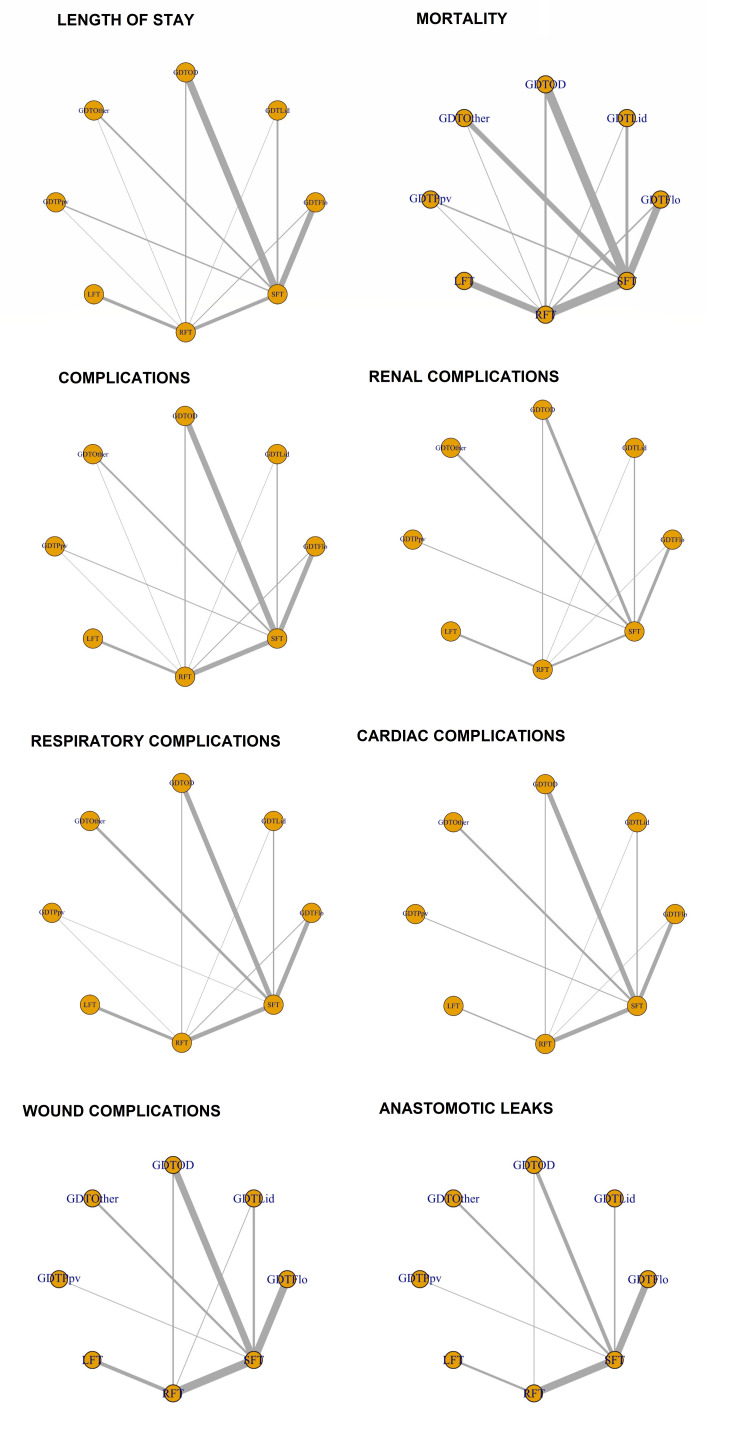
Network geometry plots for the different outcomes Each yellow dot represents a node for each intervention. The grey lines represent direct comparisons between the interventions. The thickness of the grey line represents the number of studies and patients available for that direct comparison. GDT, goal-directed therapy; GDTOD, GDT utilising oesophageal Doppler; GDTFlo, GDT using Vigileo/FloTrac; GDTLid, GDT utilising LiDCO; GDTPpv, GDT utilising pulse pressure variation or pulse variability index; GDTOthers, GDT utilising other technology; LFT, liberal fluid therapy; SFT, standard fluid therapy; RFT, restricted fluid therapy

The network geometry plots for the different outcomes are compiled and shown in Figure 2. After exclusion, 63 studies (10,608 patients) that reported LOS, 64 studies (11,219 patients) that reported mortality, 58 studies (7,591 patients) that reported complication rate, 54 studies (10,221 patients) that reported respiratory complications, 38 studies (8,803 patients) that reported renal complications, 47 studies (6,847 patients) that reported cardiac complications, 45 studies (9,335 patients) that reported wound complications and 34 studies (7,957 patients) that reported anastomotic leak were included in individual NMAs. All networks contained eight nodes. GDTFlo and GDTOD studies were the most common in most of the networks. The potential scale reduction factor (PSRF) on all the network models was less than 1.05, indicating sufficient convergence and reliability of the model. The Gelman plots and convergence plots can be obtained on request from the primary author (YX).

Risk of Bias Within Studies

**Figure 3:**
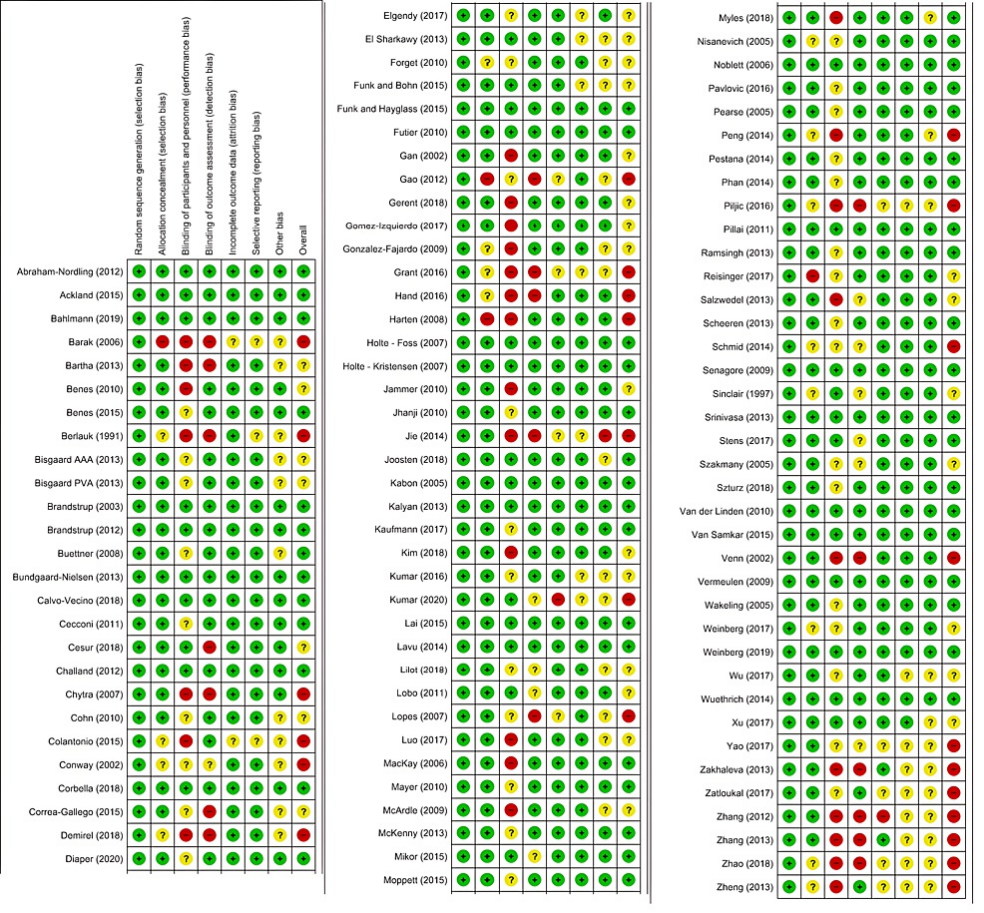
Risk of bias summary Results of risk of bias assessment representing all of the judgements in a cross-tabulation of study by entry. Green positives represent low risk of bias, yellow question marks represent unclear risk of bias and red negatives represent high risk of bias.

**Figure 4:**
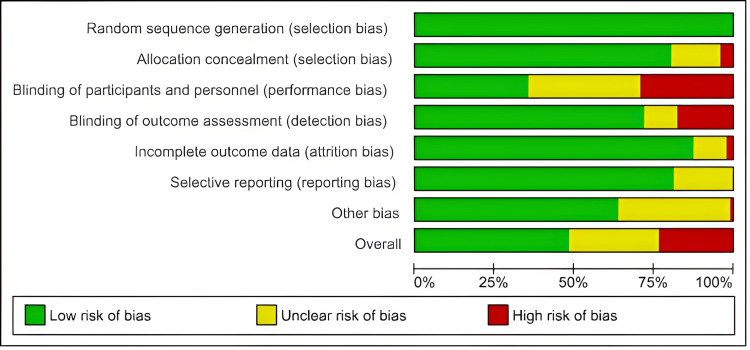
Risk of bias graph The proportion of studies with each of the judgements of risk of bias. Green represents ‘low risk’, red represents ‘high risk’ and yellow represents ‘unclear risk’ of bias.

The risks of bias within the individual studies are presented in the Appendices. The risk of bias summary is presented in Figure 3, and the risk of bias graph is presented in Figure 4.

Heterogeneity and Risk of Bias Across Studies

**Figure 5:**
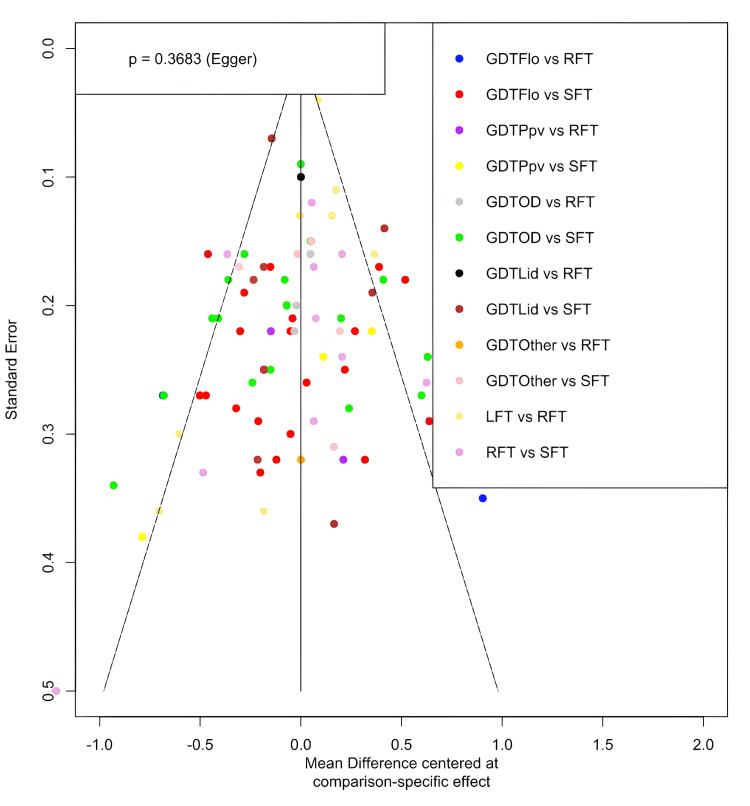
Comparison-adjusted funnel plot for the length of stay A scatterplot of the standard error of each study against the mean difference of each study for the length of stay. The solid line represents the null hypothesis that the study-specific effect sizes do not differ from the respective comparison-specific pooled effect estimates. Each coloured dot represents one study. GDT, goal-directed therapy; GDTOD, GDT utilising oesophageal Doppler; GDTFlo, GDT using Vigileo/FloTrac; GDTLid, GDT utilising LiDCO; GDTPpv, GDT utilising pulse pressure variation or pulse variability index; GDTOthers, GDT utilising other technology; LFT, liberal fluid therapy; SFT, standard fluid therapy; RFT, restricted fluid therapy

There was moderate to substantial heterogeneity in all the network meta-analyses (length of stay: I^2^ = 70.2%, moderate; mortality: I^2^ = 0%, low; complication rate: I^2^ = 78.7%, substantial; renal complications: I^2^ = 51.2%, moderate; respiratory complications: I^2^ = 49.9%, moderate; cardiac complications: I^2^ = 55.3%, moderate; wound complications: I^2^ = 28.5%, moderate; anastomotic leak complications: I^2^ = 38.9%, moderate). Therefore, random effects models were used for all the network meta-analyses. The comparison-adjusted funnel plots were found to be symmetrical. An example is produced for the length of stay (tau^2^ = 0.0838, tau = 0.2895). Egger’s test was P = 0.3683, suggesting a lack of publication bias (Figure 5).

Synthesis of Results

**Figure 6:**
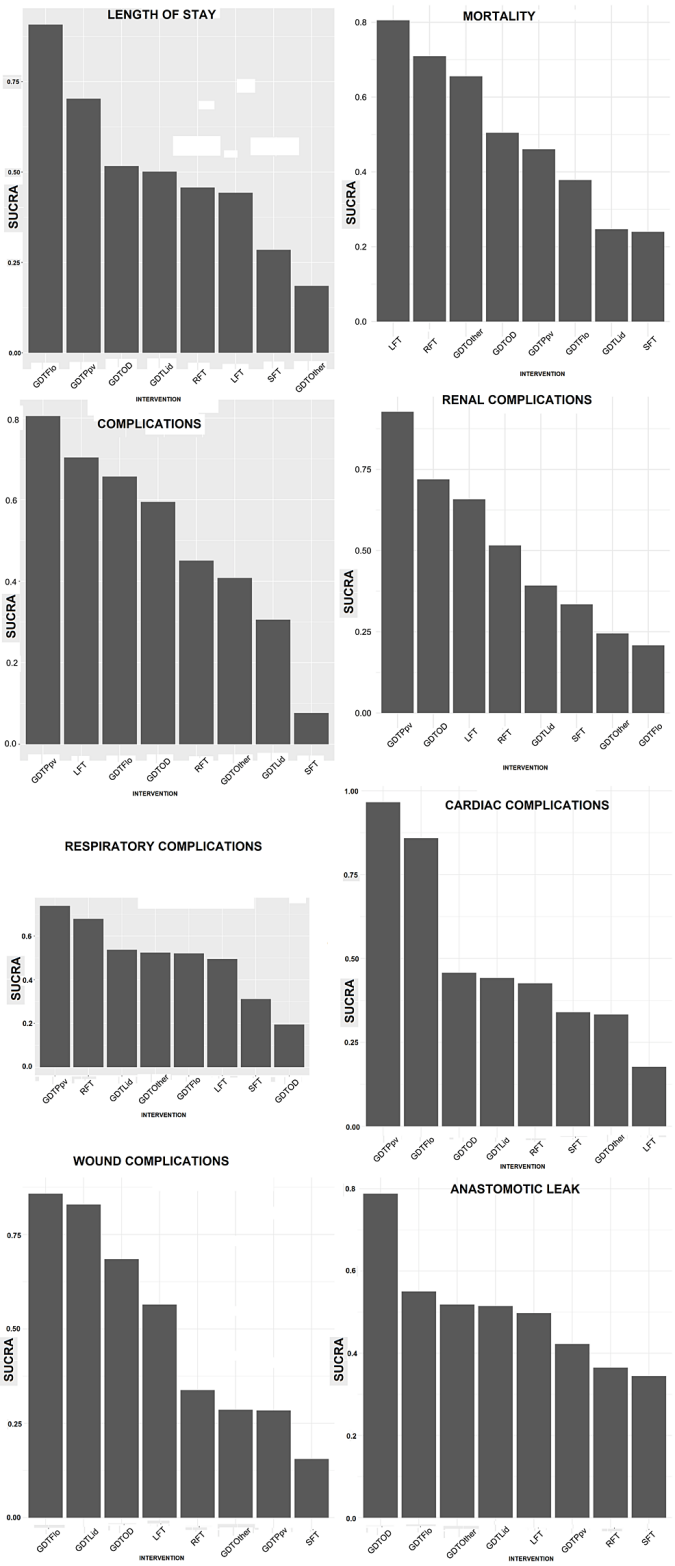
Graphs of SUCRA score (y-axis) for each intervention (x-axis) for each complication SUCRA, surface under cumulative ranking curve; goal-directed therapy, GDT; GDTOD, GDT utilising oesophageal Doppler; GDTFlo, GDT using Vigileo/FloTrac; GDTLid, GDT utilising LiDCO; GDTPpv, GDT utilising pulse pressure variation or pulse variability index; GDTOthers, GDT utilising other technology; LFT, liberal fluid therapy; SFT, standard fluid therapy; RFT, restricted fluid therapy

**Figure 7:**
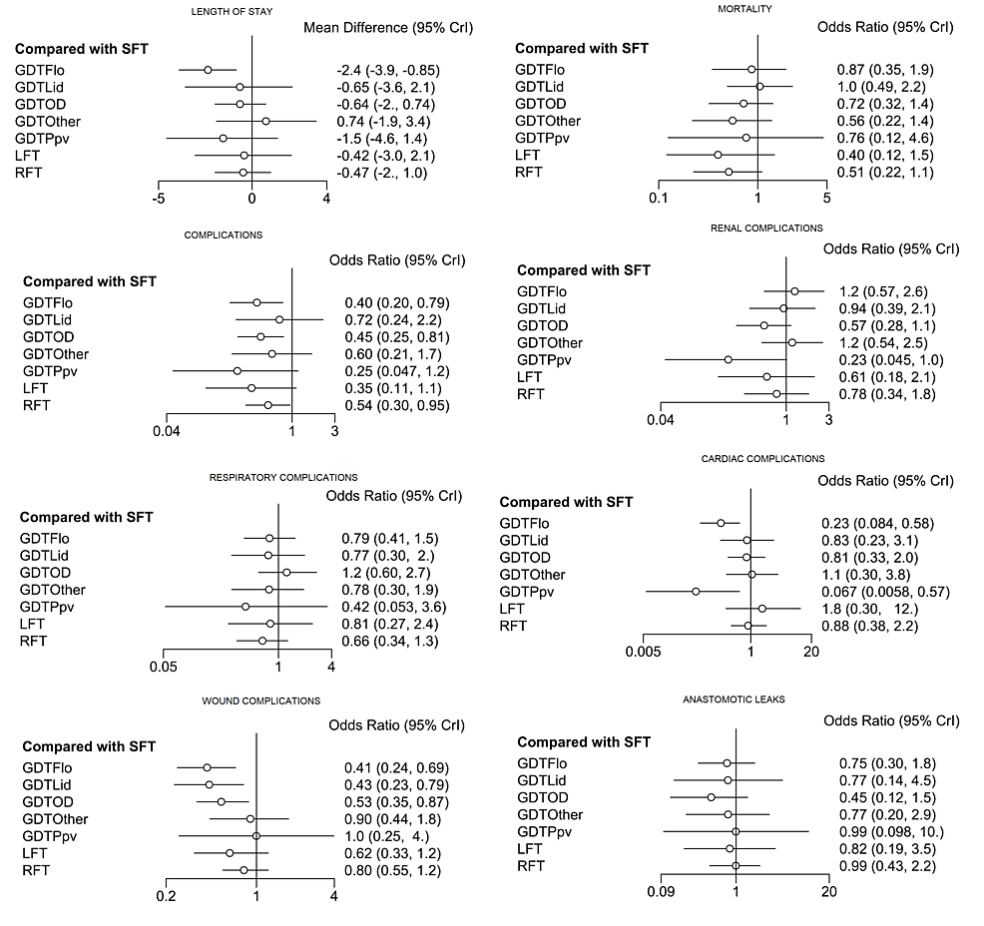
Relative effects forest plots Forest plots showing the estimated effect (mean difference) of each intervention as compared to SFT, as well as the 95% CrI for each comparison. CrI, credible interval; GDT, goal-directed therapy; GDTOD, GDT utilising oesophageal Doppler; GDTFlo, GDT using Vigileo/FloTrac; GDTLid, GDT utilising LiDCO; GDTPpv, GDT utilising pulse pressure variation or pulse variability index; GDTOthers, GDT utilising other technology; LFT, liberal fluid therapy; SFT, standard fluid therapy; RFT, restricted fluid therapy

The results of the different NMAs for the different outcome measures are summarised below (Figure 6 and Figure 7).

Length of stay: For the length of stay (LOS), the NMA suggested that GDTFlo was the most effective intervention in reducing LOS (SUCRA = 91%, OR = -2.4, 95% CrI = -3.9 to -0.85, low quality), followed by GDTPpv (SUCRA = 70%, OR = -1.5, 95% CrI = -4.6 to 1.4, very low quality) and GDTOD (SUCRA = 52%, OR = -0.64, 95% CrI = -2 to 0.74, low quality).

Mortality: LFT was the most effective intervention in reducing mortality (SUCRA = 81%, OR = 0.40, 95% CrI = 0.12 to 1.5, very low quality), followed by RFT (SUCRA = 71%, OR = 0.51, 95% CrI = 0.22 to 1.1, low quality) and GDTOther (SUCRA = 66%, OR = 0.56, 95% CrI = 0.22 to 1.4, very low quality).

Complication rate: GDTPpv was the most effective intervention in reducing complication rates (SUCRA = 80%, OR = 0.25, 95% CrI = 0.047 to 1.2, very low quality), followed by LFT (SUCRA = 70%, OR = 0.35, 95% CrI = 0.11 to 1.1, very low quality), and GDTFlo (SUCRA = 66%, OR = 0.40, 95% CrI = 0.2 to 0.79, very low quality).

Renal complications: GDTPpv was the most effective intervention in reducing renal complication (SUCRA = 93%, OR = 0.23, 95% CrI = 0.045, to 1.0, very low quality), followed by GDTOD (SUCRA = 72%, OR = 0.57, 95% CrI = 0.28 to 1.1, moderate quality) and LFT (SUCRA = 66%, OR = 0.61, 95% CrI = 0.18 to 2.1, moderate quality).

Respiratory complications: GDTPpv was the most effective intervention in reducing respiratory complications (SUCRA = 74%, OR = 0.42, 95% CrI = 0.053 to 3.6, low quality), followed by RFT (SUCRA = 68%, OR = 0.66, 95% CrI = 0.34 to 1.3, moderate quality) and GDTLid (SUCRA = 54%, OR = 0.77, 95% CrI = 0.30 to 2.0, low quality).

Cardiac complications: GDTPpv was the most effective intervention in reducing cardiac complications (SUCRA = 97.57064%, OR = 0.067, 95% CrI = 0.0058 to 0.57, very low quality), followed by GDTFlo (SUCRA = 86%, OR = 0.23, 95% CrI = 0.084 to 0.58, low quality) and GDTOD (SUCRA = 46%, OR = 0.81, 95% CrI = 0.33 to 2.0, moderate quality).

Wound complications: GDTFlo was the most effective intervention in reducing wound complications (SUCRA = 86%, OR = 0.41, 95% CrI = 0.24 to 0.69, high quality), GDTLid (SUCRA = 83%, OR = 0.43, 95% CrI = 0.23 to 0.79, high quality) and GDTOD (SUCRA = 68%, OR = 0.53, 95% CrI = 0.35 to 0.87, high quality).

Anastomotic leak: GDTOD was the most effective intervention in reducing anastomotic leaks (SUCRA = 79%, OR = 0.45, 95% CrI = 0.12 to 1.5, moderate quality), followed by GDTFlo (SUCRA = 55%, OR = 0.75, 95% CrI = 0.3 to 1.8, high quality) and GDTOther (SUCRA = 52%, OR = 0.77, 95% CrI = 0.20 to 2.9, low quality).

Exploration of Inconsistency and Additional Analyses

The following is a summary of the exploration of the inconsistency of the NMAs of the outcomes. The network summaries, full node-splitting analysis of inconsistency and the forest plots of the network meta-regression for each outcome can be obtained upon request to the primary author (YX).

Among the 12 comparisons, inconsistency was found in GDTFlo versus RFT and GDTFlo versus SFT in LOS (P = 0.002, P = 0.002), mortality, complication rate (P = 0.007, P = 0.008), renal complications (P = 0.029, P = 0.039) and cardiac complications (P = 0.006, P = 0.007) analysis. RFT versus GDTPpv and SFT versus GDTPpv (P = 0.018, P = 0.017) were inconsistent in respiratory complication analysis. RFT versus SFT was inconsistent in mortality analysis (P = 0.05). No inconsistency was found in the analysis of wound complications and anastomotic leak.

Sensitivity analyses were performed and identified that the trials of Colantonio et al. [50] and Elgendy et al. [57] contributed to the inconsistency in the comparison of complication rate and cardiac complications. The randomized controlled trial by Zatloukal et al. [128] was the source of inconsistency in renal complications analysis. The studies of Zhang et al. [130] and Lopes et al. [87] were the sources of inconsistency in respiratory complications. After removing the inconsistency, rankings remained the same or slightly altered, except in the respiratory complication analysis. RFT was found to be the most effective treatment (71%), followed by GDTLid (59%) and GDTOther (58%).

Network meta-regression was performed and showed a minimal effect of risk of bias in all analyses (Appendices).

Discussion

Summary of Evidence

This systematic review encompassed 102 studies of 14,017 patients. Seventy-eight studies involving 12,100 patients were included in this NMA. Only randomised controlled trials (RCTs) of adult patients undergoing elective non-vascular abdominal surgery were included, partially fulfilling the transitivity assumption, and there were minimal effects on the risk of bias. There was significant heterogeneity in the studies reflecting the diversity of the results, and no publication bias was detected.

The effects of GDT for wound complications and anastomotic leak are the most reliable. The evidence is high to moderate quality, with no inconsistency, minimal imprecision and indirectness, and not affected by bias. Overall, different forms of GDT were more highly ranked as interventions for the different outcomes, except for mortality.

No specific studies could be attributed to the inconsistency in LOS and mortality networks. Hence, the evidence for LOS and mortality has to be interpreted cautiously and considered to be low quality and very low quality. This could be due to low mortality in elective abdominal surgery (266 of 11,219 patients, 2.37%). Both LFT and RFT were highly ranked, which seems counterintuitive. GDTPpv has a seemingly impressive result for complication rates, renal complications, and respiratory complications but can be exaggerated by the small number of studies [3]. This highlights that GDTPpv is lacking in research and should be explored further. Pulse pressure variation and pulse variability index are promising modalities of haemodynamic monitoring that require neither expensive nor invasive equipment. The effects of GDTFlo for cardiac complications, while significant, are likely overstated.

Regarding NMA on GDT in surgical patients, our results are consistent with the findings of Zhao et al. [10]. Compared with Zhao et al. [10], we also included LFT and RFT in the current NMA with improved transitivity by only including non-vascular abdominal surgery in adults. Our study had a broader and better-defined classification system, which was based on the haemodynamic monitor used and fluid protocol in each trial. Hence, there were more studies pooled within each stratum for analysis. Also, more outcomes were measured in the current study.

Limitations

As expected, there was significant heterogeneity, a reflection of the multiple intervention arms, with a majority of studies having small sample sizes and statistically non-significant results. The lack of standardised protocols for controls made comparisons difficult as SFT differed from centre to centre. Also, several GDT studies espoused RFT principles. Future analysis may consider classifying the arms into the actual volume of fluid received instead of the haemodynamic monitor used or the authors’ intentions. The populations and types of major non-vascular abdominal surgeries between different RCTs were different. The composite outcomes utilised may have introduced bias. Sensitivity analysis was used to identify inconsistency, but it may introduce selection bias by excluding articles with contradicting data or large effect sizes. Thus, we reported the original results of the network plot with the inconsistencies found.

## Conclusions

 

In adult patients undergoing major non-vascular abdominal surgery, the use of different haemodynamic monitors and methods of GDT have different outcome-dependent effectiveness on the length of stay, mortality and different perioperative complications. GDT seems superior to either RFT or LFT in reducing perioperative complications, although the evidence is mostly low quality. The evidence was the strongest and most reliable in the outcomes of wound infections and anastomotic leak, graded as medium to high quality.

## Figures and Tables

**Figure 8:**
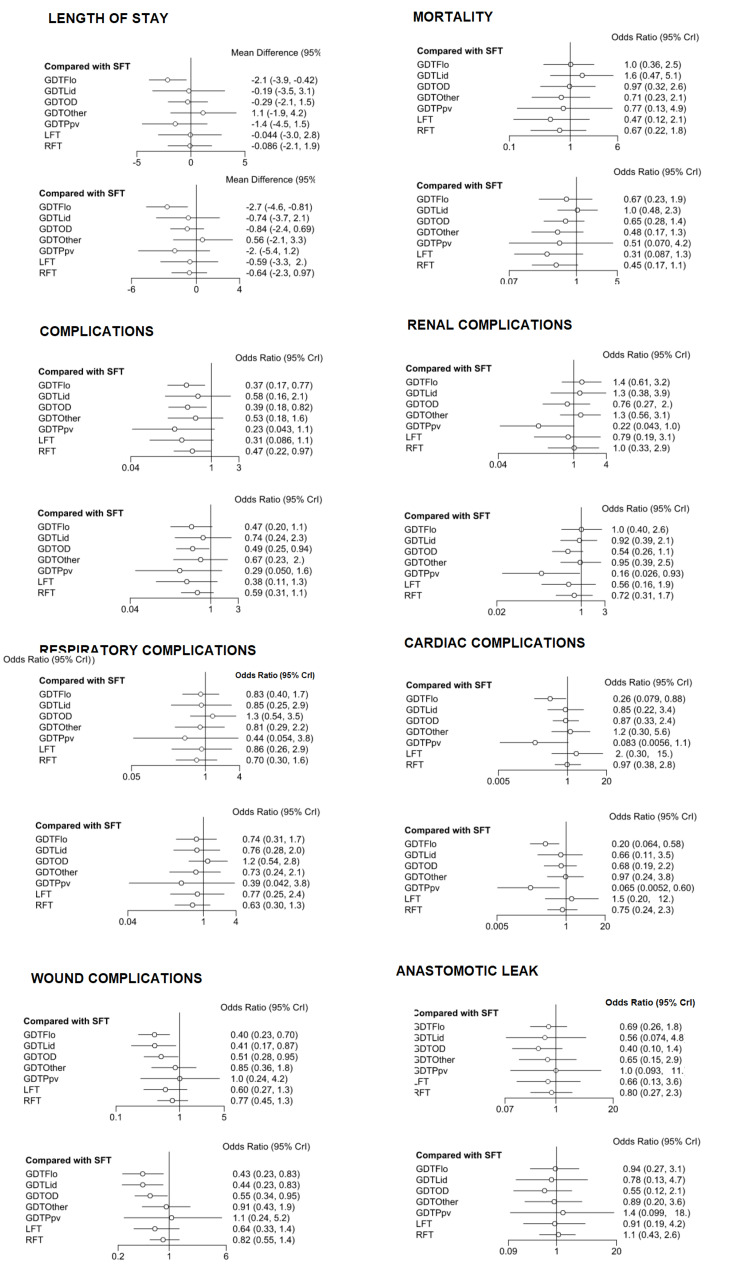
Network meta-regression Network meta-regression forest plots showing the estimated effect (mean difference) of each intervention as compared to SFT, as well as the 95% CrI for each comparison for high risk of bias studies removed versus when they were included. CrI, credible intervals; GDT, goal-directed therapy; GDTOD, GDT utilising oesophageal Doppler; GDTFlo, GDT using Vigileo/FloTrac; GDTLid, GDT utilising LiDCO; GDTPpv, GDT utilising pulse pressure variation or pulse variability index; GDTOthers, GDT utilising other technology; LFT, liberal fluid therapy; SFT, standard fluid therapy; RFT, restricted fluid therapy

## Appendices

Table 1 shows the excluded studies and their reasons for exclusion. 

**Table 1:** Excluded studies and reasons for exclusion PA, pulmonary artery; RCT, randomised controlled trial; TEE, transesophageal echocardiography; ERAS, enhanced recovery after surgery; PAC, pulmonary artery catheter

Excluded studies	Reasons for exclusion
Arulkumaran N, Corredor C, Hamilton MA, Ball J, Grounds RM, Rhodes A, Cecconi M. Cardiac complications associated with goal-directed therapy in high-risk surgical patients: a meta-analysis. Br J Anaesth. 2014 Apr;112(4):648-59. doi: 10.1093/bja/aet466. Epub 2014 Jan 10. PMID: 24413429.	Review article
Boland MR, Noorani A, Varty K, Coffey JC, Agha R, Walsh SR. Perioperative fluid restriction in major abdominal surgery: systematic review and meta-analysis of randomized, clinical trials. World J Surg. 2013 Jun;37(6):1193-202. doi: 10.1007/s00268-013-1987-8. PMID: 23463399.	Review article
Dushianthan A, Knight M, Russell P, Grocott MP. Goal-directed haemodynamic therapy (GDHT) in surgical patients: systematic review and meta-analysis of the impact of GDHT on post-operative pulmonary complications. Perioper Med (Lond). 2020 Oct 15;9:30. doi: 10.1186/s13741-020-00161-5. PMID: 33072306; PMCID: PMC7560066.	Review article
Giglio, M., Dalfino, L., Puntillo, F. et al. Hemodynamic goal-directed therapy and postoperative kidney injury: an updated meta-analysis with trial sequential analysis. Crit Care 23, 232 (2019). https://doi.org/10.1186/s13054-019-2516-4.	Review article
Pang Q, Liu H, Chen B, Jiang Y. Restrictive and liberal fluid administration in major abdominal surgery. Saudi Med J. 2017 Feb;38(2):123-131. doi: 10.15537/smj.2017.2.15077. PMID: 28133683; PMCID: PMC5329622.	Review article
Rahbari NN, Zimmermann JB, Schmidt T, Koch M, Weigand MA, Weitz J. Meta-analysis of standard, restrictive and supplemental fluid administration in colorectal surgery. Br J Surg. 2009 Apr;96(4):331-41. doi: 10.1002/bjs.6552. PMID: 19283742.	Review article
Som A, Maitra S, Bhattacharjee S, Baidya DK. Goal directed fluid therapy decreases postoperative morbidity but not mortality in major non-cardiac surgery: a meta-analysis and trial sequential analysis of randomized controlled trials. J Anesth. 2017 Feb;31(1):66-81. doi: 10.1007/s00540-016-2261-7. Epub 2016 Oct 13. PMID: 27738801.	Review article
Wrzosek A, Jakowicka-Wordliczek J, Zajaczkowska R, Serednicki WT, Jankowski M, Bala MM, Swierz MJ, Polak M, Wordliczek J. Perioperative restrictive versus goal-directed fluid therapy for adults undergoing major non-cardiac surgery. Cochrane Database Syst Rev. 2019 Dec 12;12(12):CD012767. doi: 10.1002/14651858.CD012767.pub2. PMID: 31829446; PMCID: PMC6953415.	Review article
Xu C, Peng J, Liu S, Huang Y, Guo X, Xiao H, Qi D. Goal-directed fluid therapy versus conventional fluid therapy in colorectal surgery: A meta analysis of randomized controlled trials. Int J Surg. 2018 Aug;56:264-273. doi: 10.1016/j.ijsu.2018.06.034. Epub 2018 Jul 1. PMID: 29972762.	Review article
Zhao X, Tian L, Brackett A, Dai F, Xu J, Meng L. Classification and differential effectiveness of goal-directed hemodynamic therapies in surgical patients: A network meta-analysis of randomized controlled trials. J Crit Care. 2021 Feb;61:152-161. doi: 10.1016/j.jcrc.2020.10.031. Epub 2020 Nov 3. PMID: 33171332.	Review article
Zhao X, Zhang L, Brackett A, Dai F, Xu J, Meng L. Hemodynamic management and surgical site infection: Network meta-analysis of randomized controlled trials. J Clin Anesth. 2020 Dec;67:110021. doi: 10.1016/j.jclinane.2020.110021. Epub 2020 Sep 11. PMID: 32927235.	Review article
Ashok V, Bala I, Bharti N, Jain D, Samujh R. Effects of intraoperative liberal fluid therapy on postoperative nausea and vomiting in children-A randomized controlled trial. Paediatr Anaesth. 2017 Aug;27(8):810-815. doi: 10.1111/pan.13179. Epub 2017 Jun 6. PMID: 28585750.	Pediatric
Berlauk JF, Abrams JH, Gilmour IJ, O'Connor SR, Knighton DR, Cerra FB. Preoperative optimization of cardiovascular hemodynamics improves outcome in peripheral vascular surgery. A prospective, randomized clinical trial. Ann Surg. 1991 Sep;214(3):289-97; discussion 298-9. doi: 10.1097/00000658-199109000-00011. PMID: 1929610; PMCID: PMC1358649.	PA catheter, not fluid versus fluid
Bishop MH, Shoemaker WC, Appel PL, Meade P, Ordog GJ, Wasserberger J, Wo CJ, Rimle DA, Kram HB, Umali R, et al. Prospective, randomized trial of survivor values of cardiac index, oxygen delivery, and oxygen consumption as resuscitation endpoints in severe trauma. J Trauma. 1995 May;38(5):780-7. doi: 10.1097/00005373-199505000-00018. PMID: 7760409.	Trauma resuscitation
Boyd O, Grounds RM, Bennett ED. A randomized clinical trial of the effect of deliberate perioperative increase of oxygen delivery on mortality in high-risk surgical patients. JAMA. 1993 Dec 8;270(22):2699-707. PMID: 7907668.	O2 delivery trial, PA catheter
Bloom M, Cuff G, Plichta A. Goal‐directed fluid therapy in neurosurgical cases. Journal of Neurosurgical Anesthesiology 2015;27(4):424‐5.	Abstract only
Calvo Vecino JM, Ripolles Melchor J, Martinez Hurtado E, Abad Gurumeta A, Casans Frances R, Serrano A. Efficacy of intraoperatory optimisation of fluids guided with transoesophageal doppler monitorisation: a multicentre randomised controlled trial. European Journal of Anaesthesiology2014; Vol. 31:13.	Abstract only
Chattopadhyay S, Patel A, Mittal S, Biliatis I, Christian S, Terblanche A, et al. The role of intra‐operative fluid optimisation using oesophageal doppler in advanced ovarian cancer; early postoperative recovery and fitness for discharge. International Journal of Gynecologic Cancer Volume: 23 Issue 1 (2013) ISSN: 1048-891X Online ISSN: 1525-1438	Not an RCT
Shin CH, Long DR, McLean D, Grabitz SD, Ladha K, Timm FP, Thevathasan T, Pieretti A, Ferrone C, Hoeft A, Scheeren TWL, Thompson BT, Kurth T, Eikermann M. Effects of Intraoperative Fluid Management on Postoperative Outcomes: A Hospital Registry Study. Ann Surg. 2018 Jun;267(6):1084-1092. doi: 10.1097/SLA.0000000000002220. PMID: 28288059.	Not an RCT
Concha PM, Mertz KV, Cortínez FL, Zúñiga DA, Pinedo MG. Transesophageal echocardiography to monitor fluid administration during the perioperative period. Revista Medica de Chile 2011;139(9):1157‐62. (PUBMED: 22215394).	Non-English
Cordero‐Rochet MJ, McCluskey SA, Minkovich L, Gilbert R. Goal directed fluid management in free flap reconstructive surgery. Canadian Journal of Anesthesia 2014;61:S75.	Abstract only
Donati A, Loggi S, Preiser JC, Orsetti G, Münch C, Gabbanelli V, Pelaia P, Pietropaoli P. Goal-directed intraoperative therapy reduces morbidity and length of hospital stay in high-risk surgical patients. Chest. 2007 Dec;132(6):1817-24. doi: 10.1378/chest.07-0621. Epub 2007 Oct 9. PMID: 17925428.	Oxygen delivery-focused, not fluid therapy
Dhawan R, Shahul S, Roberts JD, Smith ND, Steinberg GD, Chaney MA. Prospective, randomized clinical trial comparing use of intraoperative transesophageal echocardiography to standard care during radical cystectomy. Annals of Cardiac Anaesthesia 2018;21(3):255‐61. (PUBMED: 30052211).	Did not report primary or secondary end points
Foppa C, Zakhaleva J, Tam J, Denoya PI, Bishawi M, Bergamaschi R. The impact of intravenous fluid administration on complication rates in bowel surgery with enhanced recovery protocol: a randomized controlled trial. Techniques in Coloproctology 2014;18(1):91.	Similar to the 2013 paper of Zakhaleva et al. [127]
Fischer M, Matsuo K, Gonen M, Grant F, Dematteo RP, D'Angelica MI, Mascarenhas J, Brennan MF, Allen PJ, Blumgart LH, Jarnagin WR. Relationship between intraoperative fluid administration and perioperative outcome after pancreaticoduodenectomy: results of a prospective randomized trial of acute normovolemic hemodilution compared with standard intraoperative management. Ann Surg. 2010 Dec;252(6):952-8. doi: 10.1097/SLA.0b013e3181ff36b1. PMID: 21107104.	Acute normovolemic haemodilution
Forget P, Lois F, De Kock M. Does the pleth variability index improve fluid management during major abdominal surgery?. Critical Care (London, England) 2009;13(Suppl 1):P204.	Poster of the 2010 paper of Forget et al. [58]
Forget P, Lois F, Kartheuser A, Leonard D, Remue C, Kock M. The concept of titration can be transposed to fluid management. But does it change the volumes? Randomised trial on pleth variability index during fast‐track colonic surgery. Current Clinical Pharmacology2013; Vol. 8, issue 2:110‐4. (PUBMED: 23061978).	Too similar to primary authors’ previous paper, possible overlap of patient cohort
Fukui K, Markstaller K, Leibundgut D, Pestel G. Timing of intraoperative fluid management by difference in pulse pressure (dPP). European Journal of Anaesthesiology 2009;45:68.	Abstract only
Funcke S, Saugel B, Koch C, Schulte D, Zajonz T, Sander M, et al. Individualized, perioperative, hemodynamic goal‐directed therapy in major abdominal surgery (iPEGASUS trial): study protocol for a randomized controlled trial. Trials 2018;19(1):273. (PUBMED: 29743101).	Protocol only
Goepfert MS, Richter HP, Zu Eulenburg C, Gruetzmacher J, Rafflenbeul E, Roeher K, von Sandersleben A, Diedrichs S, Reichenspurner H, Goetz AE, Reuter DA. Individually optimized hemodynamic therapy reduces complications and length of stay in the intensive care unit: a prospective, randomized controlled trial. Anesthesiology. 2013 Oct;119(4):824-36. doi: 10.1097/ALN.0b013e31829bd770. PMID: 23732173.	Cardiac surgery study
Gottin, L., Martini, A., Menestrina, N. et al. Perioperative Fluid Administration in Pancreatic Surgery: a Comparison of Three Regimens. J Gastrointest Surg 24, 569–577 (2020). https://doi.org/10.1007/s11605-019-04166-4.	Non-randomized (prospective study)
Hendrix RJ, Damle A, Williams C, Harris A, Spanakis S, Lambert DH, Lambert LA. Restrictive Intraoperative Fluid Therapy is Associated with Decreased Morbidity and Length of Stay Following Hyperthermic Intraperitoneal Chemoperfusion. Ann Surg Oncol. 2019 Feb;26(2):490-496. doi: 10.1245/s10434-018-07092-y. Epub 2018 Dec 4. PMID: 30515670.	Retrospective review
Hughes T, Cottam S, Heaton N, Bernal W, Auzinger G, Wendon J, et al. Peri‐operative haemodynamic optimisation using Pulsioflex monitoring in Whipple's surgery. Anaesthesia 2013;68:33.	Non-randomized
Jammer I, Tuovila M, Ulvik A. Stroke volume variation to guide fluid therapy: is it suitable for high-risk surgical patients? A terminated randomized controlled trial. Perioper Med (Lond). 2015 Jul 22;4:6. doi: 10.1186/s13741-015-0016-x. PMID: 26203353; PMCID: PMC4511544.	Trial terminated
Johnson E, Nunoo R, Al‐Abbad J, Senagore A, Emery T, Dujovny N, et al. Intraoperative fluid management and its correlation with the surgical Apgar score in laparoscopic segmental colectomy. Diseases of the Colon and Rectum 2011;54(5):e200.	Abstract
Kapoor PM, Kakani M, Chowdhury U, Choudhury M, Lakshmy, Kiran U. Early goal-directed therapy in moderate to high-risk cardiac surgery patients. Ann Card Anaesth. 2008 Jan-Jun;11(1):27-34. doi: 10.4103/0971-9784.38446. PMID: 18182756.	Cardiac surgery
Kellman S, Roberts JD, Chaney M, Negron E. Prospective randomized clinical trial comparing routine intraoperative transesophageal echocardiography to standard care during radical cystectomy. Anesthesia and Analgesia 2014;118(5 Suppl 1):S53.	TEE study not fluid study, abstract only
Kulkarni R, Craske DA, Abdel‐Galil K, Hatfield A, Liu A, Pick A, et al. Haemodynamic optimisation in head and neck cancer surgery: pilot randomised controlled trial of LiDCO rapid. European Archives of Oto‐Rhino‐Laryngology 2012;269(4):1370.	Abstract only
Lahtinen SL, Liisanantti JH, Poukkanen MM, Laurila PA. Goal-directed fluid management in free flap surgery for cancer of the head and neck. Minerva Anestesiol. 2017 Jan;83(1):59-68. doi: 10.23736/S0375-9393.16.11451-8. Epub 2016 Oct 19. PMID: 27759740.	Retrospective analysis
Li H, Afzal A, Lian Q, Kramer GC, Svensen C, Prough D. Restricted fluid therapy decreases surgical blood loss ‐ a clinical study of two fluid regimens during cesarean section under spinal anesthesia. Anesthesia and Analgesia 2011;112(5 Suppl 1):291.	Abstract only
Liu TJ, Zhang JC, Gao XZ, Tan ZB, Wang JJ, Zhang PP, et al. Clinical research of goal‐directed fluid therapy in elderly patients with radical resection of bladder cancer. Journal of Cancer Research and Therapeutics 2018;14(Suppl 1):S173‐9. (PUBMED: 29578169).	Case-control study, non-randomized
Martini A, Menestrina N, Simion D, Filetici L, Schweiger V, Gottin L. Perioperative fluid administration in pancreatic surgery: comparison of three regimens. Critical Care 2009;13:S80‐1.	Interim analysis of the study of Gottin et al.
McArdle GT, Price G, Lewis A, Hood JM, McKinley A, Blair PH, Harkin DW. Positive fluid balance is associated with complications after elective open infrarenal abdominal aortic aneurysm repair. Eur J Vasc Endovasc Surg. 2007 Nov;34(5):522-7. doi: 10.1016/j.ejvs.2007.03.010. Epub 2007 Sep 6. PMID: 17825590.	Retrospective cohort study
Mccaul, James & Sutton, D.N. & Hatfield, A. & Pick, A. & Liu, A. & Craske, D.C. (2011). P243. Randomised controlled trial of LidCO rapid goal directed therapy versus control for fluid optimisation in patients undergoing major head and neck cancer surgery. Oral Oncology - ORAL ONCOL. 47. 10.1016/j.oraloncology.2011.06.486.	Abstract only
McKendry M, McGloin H, Saberi D, Caudwell L, Brady AR, Singer M. Randomised controlled trial assessing the impact of a nurse delivered, flow monitored protocol for optimisation of circulatory status after cardiac surgery. BMJ. 2004 Jul 31;329(7460):258. doi: 10.1136/bmj.38156.767118.7C. Epub 2004 Jul 8. Erratum in: BMJ. 2004 Aug 21;329(7463):438. PMID: 15242867; PMCID: PMC498021.	Cardiac surgery
Melis M, Marcon F, Masi A, Sarpel U, Miller G, Moore H, Cohen S, Berman R, Pachter HL, Newman E. Effect of intra-operative fluid volume on peri-operative outcomes after pancreaticoduodenectomy for pancreatic adenocarcinoma. J Surg Oncol. 2012 Jan;105(1):81-4. doi: 10.1002/jso.22048. Epub 2011 Jul 25. PMID: 21792977.	Not an RCT
Minkovich L, McCluskey SA, Djaiani G, Goldstein D, Gilbert R. Goal directed fluid therapy in surgery for head and neck cancers. Canadian Journal of Anesthesia 2012;59:1344381.	Abstract only
Minto G, Challand C, Sneyd JR, Mellor N, Hosie KB, Erasmus P, et al. Is the impact of intraoperative goal‐directed fluid therapy on length of stay after major elective colorectal surgery related to patients' aerobic fitness?. British Journal of Anaesthesia 2011;106(3):440.	Abstract only
Mintz Y, Weiss YG, Rivkind AI. Effects of intravenous fluid restriction on postoperative complications: comparison of two perioperative fluid regimens: a randomized assessor-blinded multicenter trial. Ann Surg. 2004 Aug;240(2):386; author reply 386-8. doi: 10.1097/01.sla.0000134633.10987.87. PMID: 15273568; PMCID: PMC1356423.	Reply to the paper of Brandstrup et al. [41], correspondence
Muller S, Zalunardo MP, Hubner M, Clavien PA, Demartines N. A fast‐track program reduces complications and length of hospital stay after open colonic surgery. Gastroenterology2009; Vol. 136, issue 3:842‐7. (PUBMED: 19135997).	ERAS-focused, not comparing fluid therapy
Munoz CA, Rojas JLT, Bermudez OIG. Intraoperative oesophageal doppler during emergency abdominal surgery. British Journal of Anaesthesia 2012;108:ii347.	Not fluid therapy RCT
Mythen MG, Webb AR. Perioperative plasma volume expansion reduces the incidence of gut mucosal hypoperfusion during cardiac surgery. Arch Surg. 1995 Apr;130(4):423-9. doi: 10.1001/archsurg.1995.01430040085019. PMID: 7535996.	Cardiac study
NCT03193320. Management of intraoperative fluids in ambulatory surgery [Intraoperative fluid therapy management in low‐risk patients under general anesthesia ‐ a randomized controlled trial comparing liberal, restrictive and pleth variability index (PVI)‐guided fluid administration in a day surgery setting]. https://clinicaltrials.gov/ct2/show/record/NCT03193320 (first received 15 June 2017).	Trial protocol, not yet complete or recruiting
Osawa EA, Rhodes A, Landoni G, Galas FR, Fukushima JT, Park CH, Almeida JP, Nakamura RE, Strabelli TM, Pileggi B, Leme AC, Fominskiy E, Sakr Y, Lima M, Franco RA, Chan RP, Piccioni MA, Mendes P, Menezes SR, Bruno T, Gaiotto FA, Lisboa LA, Dallan LA, Hueb AC, Pomerantzeff PM, Kalil Filho R, Jatene FB, Auler Junior JO, Hajjar LA. Effect of Perioperative Goal-Directed Hemodynamic Resuscitation Therapy on Outcomes Following Cardiac Surgery: A Randomized Clinical Trial and Systematic Review. Crit Care Med. 2016 Apr;44(4):724-33. doi: 10.1097/CCM.0000000000001479. PMID: 26646462.	Cardiac
Park S, Kim H, Koo B. Effect of goal‐directed fluid therapy using stroke volume variation in free flap reconstruction. Anesthesia and Analgesia 2016;122(5 Suppl 3):S302.	Abstract or poster
Peng NH, Gao T, Chen YY, Xi FC, Zhang JJ, Li N, Zhu WM, Yu WK. Restricted intravenous fluid regimen reduces fluid redistribution of patients operated for abdominal malignancy. Hepatogastroenterology. 2013 Oct;60(127):1653-9. PMID: 24627921.	Not an indexed journal, article cannot be retrieved (journal discontinued)
Pösö T, Winsö O, Aroch R, Kesek D. Perioperative fluid guidance with transthoracic echocardiography and pulse-contour device in morbidly obese patients. Obes Surg. 2014 Dec;24(12):2117-25. doi: 10.1007/s11695-014-1329-4. PMID: 24902655.	Non-randomized, non-blinded
Rath G, Mishra N, Chaturvedi A, Bithal P. Effect of goal‐directed intraoperative fluid therapy on hospital stay in patients undergoing excision of supratentorial tumors. Journal of Neurosurgical Anesthesiology 2018;30(4):421‐2.	Abstract only
Rinehart J, Lilot M, Lee C, Joosten A, Huynh T, Canales C, Imagawa D, Demirjian A, Cannesson M. Closed-loop assisted versus manual goal-directed fluid therapy during high-risk abdominal surgery: a case-control study with propensity matching. Crit Care. 2015 Mar 19;19(1):94. doi: 10.1186/s13054-015-0827-7. PMID: 25888403; PMCID: PMC4372998.	Case-control study, not an RCT
Self WH, Semler MW, Bellomo R, Brown SM, deBoisblanc BP, Exline MC, Ginde AA, Grissom CK, Janz DR, Jones AE, Liu KD, Macdonald SPJ, Miller CD, Park PK, Reineck LA, Rice TW, Steingrub JS, Talmor D, Yealy DM, Douglas IS, Shapiro NI; CLOVERS Protocol Committee and NHLBI Prevention and Early Treatment of Acute Lung Injury (PETAL) Network Investigators. Liberal Versus Restrictive Intravenous Fluid Therapy for Early Septic Shock: Rationale for a Randomized Trial. Ann Emerg Med. 2018 Oct;72(4):457-466. doi: 10.1016/j.annemergmed.2018.03.039. Epub 2018 May 10. PMID: 29753517; PMCID: PMC6380679.	Not an RCT
Silversides, J.A., Perner, A. & Malbrain, M.L.N.G. Liberal versus restrictive fluid therapy in critically ill patients. Intensive Care Med 45, 1440–1442 (2019). https://doi.org/10.1007/s00134-019-05713-y.	Not a trial
Sundaram SC, Salins SR, Nandha Kumar A, Korula G. Intra‐operative fluid management in adult neurosurgical patients undergoing intracranial tumour surgery: randomised control trial comparing pulse pressure variance (PPV) and central venous pressure (CVP). Journal of Clinical and Diagnostic Research 2016;10(5):UC01‐5. (PUBMED: 27437329).	Does not assess primary or secondary end points
Ueno S, Tanabe G, Yamada H, Kusano C, Yoshidome S, Nuruki K, Yamamoto S, Aikou T. Response of patients with cirrhosis who have undergone partial hepatectomy to treatment aimed at achieving supranormal oxygen delivery and consumption. Surgery. 1998 Mar;123(3):278-86. PMID: 9526519.	Not an RCT
Valentine RJ, Duke ML, Inman MH, Grayburn PA, Hagino RT, Kakish HB, Clagett GP. Effectiveness of pulmonary artery catheters in aortic surgery: a randomized trial. J Vasc Surg. 1998 Feb;27(2):203-11; discussion 211-2. doi: 10.1016/s0741-5214(98)70351-9. PMID: 9510275.	PAC article
Vanakas T, Asouhidou I, Samaras A, Diminikos G, Ioannou P. Implementation of goal‐directed protocol in elderly patients undergoing femoral fracture repair. European Journal of Anaesthesiology2012; Vol. 29:67.	Abstract
Wang S, Wang X, Dai H, Han J, Li N, Li J. The effect of intraoperative fluid volume administration on pancreatic fistulas after pancreaticoduodenectomy. J Invest Surg. 2014 Apr;27(2):88-94. doi: 10.3109/08941939.2013.839766. PMID: 24665844.	Retrospective case-control, non-RCT
Wen XL, Jing GX, He P, Hou JR. Clinical study on the capacity management guided by stroke volume variation in elderly patients with laparoscopic radical gastrectomy for gastric cancer. Journal of Xi'an Jiaotong University (Medical Sciences) 2016;37(6):851‐6.	Non-English study
Wenkui Y, Ning L, Jianfeng G, Weiqin L, Shaoqiu T, Zhihui T, Tao G, Juanjuan Z, Fengchan X, Hui S, Weiming Z, Jie-Shou L. Restricted peri-operative fluid administration adjusted by serum lactate level improved outcome after major elective surgery for gastrointestinal malignancy. Surgery. 2010 Apr;147(4):542-52. doi: 10.1016/j.surg.2009.10.036. Epub 2009 Dec 11. PMID: 20004445.	Lactate protocol on both arms, not goal-directed therapy
Wilmin S, Dierick A, De Hert S, Van Der Linden P. Optimization of oxygen delivery in vascular surgery with Flotrac System, a prospective double blind randomized study. European Journal of Anaesthesiology 2009;45:38.	Abstract
Wilson J, Woods I, Fawcett J, Whall R, Dibb W, Morris C, et al. Reducing the risk of major elective surgery: randomised controlled trial of preoperative optimisation of oxygen delivery. BMJ 1999;318(7191):1099‐1103. (PUBMED: 10213716).	Inotrope versus inotrope study
Xiao W, Duan QF, Fu WY, Chi XZ, Wang FY, Ma DQ, et al. Goal‐directed fluid therapy may improve hemodynamic stability of parturient with hypertensive disorders of pregnancy under combined spinal epidural anesthesia for cesarean delivery and the well‐being of newborns. Chinese Medical Journal 2015;128(14):1922‐31. (PUBMED: 26168834).	Pregnant patient, caesarean section
Yin K, Ding J, Wu Y, Peng M. Goal-directed fluid therapy based on noninvasive cardiac output monitor reduces postoperative complications in elderly patients after gastrointestinal surgery: A randomized controlled trial. Pak J Med Sci. 2018 Nov-Dec;34(6):1320-1325. doi: 10.12669/pjms.346.15854. PMID: 30559778; PMCID: PMC6290223.	Too high risk of bias, likely fabricated data
Yu X, Yan J, Zhai Z, Ma X. Optimized management of fluid volume guided by PiCCO parameters in skull base tumor resection. Critical Care Medicine 2016;44(12 Suppl 1):473.	Abstract only
Yu Y, Dong J, Xu Z, Shen H, Zheng J. Pleth variability index-directed fluid management in abdominal surgery under combined general and epidural anesthesia. J Clin Monit Comput. 2015 Feb;29(1):47-52. doi: 10.1007/s10877-014-9567-5. Epub 2014 Feb 21. PMID: 24557584.	Did not report primary or secondary end points
Ziegler DW, Wright JG, Choban PS, Flancbaum L. A prospective randomized trial of preoperative "optimization" of cardiac function in patients undergoing elective peripheral vascular surgery. Surgery. 1997 Sep;122(3):584-92. doi: 10.1016/s0039-6060(97)90132-x. PMID: 9308617.	PAC study, not fluid therapy study
Zeng K, Li Y, Liang M, Gao Y, Cai H, Lin C. The influence of goal‐directed fluid therapy on the prognosis of elderly patients with hypertension and gastric cancer surgery. Drug Design, Development and Therapy2014; Vol. 8:2113‐9. (PUBMED: 25378913).	Retracted
Zheng L, Gu E, Peng X, Zhang L, Cao Y. Effect of goal‐directed haemodynamic management on the postoperative outcome in elderly patients with fragile cardiac function undergoing abdominal surgery. National Medical Journal of China2016; Vol. 96, issue 43:3464‐9. (PUBMED: 27903339).	Non-English study

Table 2 shows the included studies and their characteristics.

**Table 2:** Characteristics and primary outcomes of included studies RFT, restricted fluid therapy; SFT, standard fluid therapy; LFT, liberal fluid therapy; GDT, goal-directed therapy; R-GDT, restrictive goal-directed therapy; AAA, abdominal aortic aneurysm; postop, postoperative; EBL, estimated blood loss; LR, lactated Ringer’s solution; HAES 6%, hydroxyethyl starch 6%; NS, normal saline; SV, stroke volume; PPV, pulse pressure variation; GEDI, global end-diastolic index; ITBV, intrathoracic blood volume; FTc, flow time (corrected); RCT, randomised controlled trial; TKR, total knee replacement; THR, total hip replacement; GDTPpv, GDT utilising pulse pressure variation or pulse variability index; HIPEC, hyperthermic intraperitoneal chemotherapy; GDTFlo: GDT using Vigileo/FloTrac; LiDCO, lithium dilution cardiac output; GDTLid, GDT utilising LiDCO; SVI, stroke volume index; GDTClear, GDT utilising Clearsight System; SVV, stroke volume variation; CI, cardiac index; GDTOD, GDT utilising oesophageal Doppler; PPV, pulse pressure variation; ERAS, enhanced recovery after surgery; PAC, pulmonary artery catheter; GDTPic, GDT utilising PiCCO; PVI, pleth variability index; GDTPVI: GDT utilising pleth variability index; CVP, central venous pressure; MAP, mean arterial pressure; CO, cardiac output; Scvo2, central venous oxygen saturation; GDTScv, GDT utilising Scvo2; GDTNic: GDT utilising NICOM; GDTPro: GDT utilising ProAQT; GDTNex, GDT utilising Nexfin [2,7,30-38,40-82,84,86-132]

Author (year)	Design of the study (country)	Type of surgery	Type of intervention versus control	Phase of intervention	Fluid protocols used (intervention versus control)	Number of patients (intervention versus control)	Age of patients (intervention versus control)	In-hospital mortality	Disability-free survival at one year	Length of hospital stay (days)
RFT versus SFT										
Abraham-Nordling (2012)	Single-centre RCT (Sweden)	Elective colorectal surgery	RFT versus SFT	Intraoperative	RFT = 2 mL/kg/hour, D10, SFT = preloading, 5 mL/kg/hour and additional 1,000 mL of fluid, LR	RFT = 79, SFT = 82	RFT = 68, SFT = 69	RFT = 0, SFT = 0	Not reported	RFT = 6 (4-8), SFT = 6 (4-8), P = 0.194
Brandstrup (2003)	Multicentre RCT (Denmark)	Elective colorectal surgery	RFT versus SFT	Intraoperative	RFT = volume to volume replacement with HAES 6%, SFT = preloading, 7 mL/kg/hour for the first hour, 5 mL/kg/hour for the second and third hour, 3 mL/kg/hour following hours, NS, 2-3:1 volume replacement with NS	RFT = 69, SFT = 72	RFT = 64, SFT = 69	RFT = 0, SFT = 4, P = 0.12	Not reported	RFT = 10.8 (7.53), SFT = 12.1 (10.9)
Cohn (2010)	Single-centre RCT (USA)	Elective colorectal surgery	RFT versus SFT	Intraoperative	RFT = 2 mL/kg/hour, 1:1 fluid replacement for blood loss, LR, SFT = preloading, 7 mL/kg/hour for the first hour, followed by 5 mL/kg/hour, 3:1 fluid replacement for EBL, LR	RFT = 18, SFT = 9	RFT = 56, SFT = 45	RFT = 0, SFT = 0	Not reported	Not reported
Futier (2010)	Single-centre RCT (France)	Major abdominal surgery	RFT versus SFT	Intraoperative, postoperative	RFT = 6 mL/kg/hour LR, SFT = 12 mL/kg/hour LR	RFT = 36, SFT = 34	RFT = 61.9, SFT = 60.4	RFT = 1, SFT = 1	Not reported	Not reported
Gao (2012)	Single-centre RCT (China)	Gastrointestinal surgery	RFT versus SFT	Intraoperative, postoperative	RFT = 7 mL/kg/hour followed by 5 mL/kg/hour LR, 1,000-1,500 mL/day, SFT = preloading, 12 mL/kg/hour LR, 2,000-2,500 mL/day	RFT = 93, SFT = 86	RFT = 72, SFT = 73	RFT = 2, SFT = 4 P = 0.353	Not reported	Not reported
González-Fajardo (2009)	Single-centre RCT (Spain)	Open AAA surgery	RFT versus SFT	Postoperative	RFT = 1,500 mL/day, NS, SFT = 2,500 mL/day, dextrose and NS	RFT = 20, SFT = 20	RFT = 65.5, SFT = 61.95	RFT = 0, SFT = 1	Not reported	RFT = 8.4 (7.75-9.05), SFT = 12.4 (8.68-16.12)
Jie (2014)	Single-centre RCT (China)	Elective colorectal surgery	RFT versus SFT	Intraoperative, postoperative	RFT = no preloading, 7 mL/kg in the first hour and then 5 mL/kg in the following hours LR, 1,000-1,500 mL/day, crystalloid, SFT = preloading, 500 mL 6% HAES, 12 mL/kg/hour LR, 2,000-2,500 mL/day, crystalloid	RFT = 89, SFT = 96	RFT = 64.7, SFT = 65.4	RFT = 0, SFT = 0	Not reported	Not reported
Lavu (2014)	Single-centre RCT (USA)	Pancreatic surgery	RFT versus SFT	Intraoperative, postoperative	RFT = 1 mL/kg hypertonic saline, followed by 9 mL/kg/hour LR, EBL replaced 1:1, SFT = 15 mL/kg/hour LR, EBL replaced 3:1	RFT = 131, SFT = 128	RFT = 66.6, SFT = 68.3	RFT = 0, SFT = 1	Not reported	RFT = 7 (4-41), SFT = 7 (5-61)
Lobo (2011)	Single-centre RCT (Brazil)	High-risk elective surgery	RFT-GDT versus SFT-GDT	Intraoperative	RFT = 4 mL/kg/hour LR, LiDCO, SFT = 12 mL/kg/hour LR, LiDCO	RFT = 45, SFT = 43	RFT = 69.2, SFT = 68.6	RFT = 0, SFT = 2	Not reported	RFT = 6 (4-9), SFT = 6 (4-10)
MacKay (2006)	Single-centre RCT (UK)	Elective colorectal surgery	RFT versus SFT	Intraoperative, postoperative	RFT = 2,000 mL/day of NS + dextrose until D1, SFT = 3,000 mL/day NS + dextrose until D3	RFT = 37, SFT = 32	RFT = 73.2, SFT = 72.6	RFT = 1, SFT = 1	Not reported	RFT = 7.2 (6.1-11.2), SFT = 7.2 (6.1-11.2), P = 0.902
McArdle (2009)	Single-centre RCT (UK)	Open AAA surgery	RFT versus SFT	Intraoperative, postoperative	RFT = no preload, 4 mL/kg/hour LR, 2,000 mL/day crystalloid + dextrose, SFT = 10 mL/kg NS preload, 12 mL/kg/hour LR, 3,000 mL/day crystalloid + dextrose	RFT = 9, SFT = 11	RFT = 74, SFT = 75	RFT = 0, SFT = 0	Not reported	RFT = 7.78 (+/- 0.64), SFT = 16 (+/- 4.82), P < 0.025
Piljic (2016)	Multicentre RCT (Bosnia)	Open AAA surgery	RFT versus SFT	Intraoperative, postoperative	RFT = 10 mL/kg/hour LR, postoperative 70-100 mL/hour, SFT = 15 mL/kg/hour LR, postoperative 150-200 mL/hour	RFT = 30, SFT = 30	RFT = 68.64, SFT =69.34	RFT = 0, SFT = 1	Not reported	RFT = 4.33, SFT = 6.20, P = 0.035
Van Samkar (2015)	Single-centre RCT (Netherlands)	Pancreatic surgery	RFT versus SFT	Intraoperative	RFT = 5 mL/kg/hour LR, SFT = 10 mL/kg/hour LR, postoperative standardized	RFT = 26, SFT = 22	No significant difference	RFT = 1, SFT = 1	Not reported	RFT = 12 (11-14), SFT = 10 (9-17)
Vermeulen (2009)	Single-centre RCT (Netherlands)	Major abdominal surgery	RFT versus SFT	Postoperative	RFT = 1,500 mL/day, SFT = 2,500 mL/day	RFT = 30, SFT = 32	RFT = 55.5, SFT = 53.6	RFT = 1, SFT = 0	Not reported	RFT = 12.3 (12.7), SFT = 8.3 (4.5), P = 0.049
Wuethrich (2014)	Single-centre RCT (Switzerland)	Radical cystectomy	RFT versus SFT	Intraoperative	RFT = 1 mL/kg/hour, followed by 3 mL/kg/hour LR, SFT = 6 mL/kg/hour LR	RFT = 83, SFT = 83	RFT = 68, SFT = 69	RFT = 0, SFT = 4	Not reported	RFT = 15 (11-27), SFT = 17 (10-95), P = 0.01
RFT versus GDT										
Benes (2015)	Single-centre RCT (Czech Republic)	THR and TKR (orthopaedic surgery)	RFT versus GDTPpv versus SFT	Intraoperative	RFT = 5 mL/kg/hour crystalloid, GDT = 5 mL/kg/hour crystalloid, PPV directed, SFT = no protocol	RFT = 40, GDT = 40, SFT = 40	RFT = 66, GDT = 68, SFT = 70	RFT = 1, GDT = 0, SFT = 0	Not reported	RFT = 10 (9-12.5), GDT = 10 (8.5-13.5), SFT = 10.5 (8-12), P = 0.99
Brandstrup (2012)	Multicentre RCT (Denmark)	Elective colorectal surgery	RFT versus GDTOD	Intraoperative	RFT = HAES 6% to maintain zero balance, GDT = oesophageal Doppler, fluid boluses until increase in SV < 10%	RFT =79, GDT = 72	RFT = 68.1, GDT = 66.9	RFT = 1, GDT = 1	Not reported	RFT = 7.66 (8.2), GDT = 8.45 (7.5), P = 0.539
Colantonio (2015)	Single-centre RCT (Italy)	Major abdominal surgery with HIPEC	RFT versus GDTFlo	Intraoperative	RFT = restrictive 4-10 mL/kg/hour, GDT = FloTrac, keep CI > 2.5 L/minute/m^2^, 4 mL/kg/hour	RFT = 44, GDT = 42	RFT = 57.6, GDT = 54.5	RFT = 4, GDT = 0, P = 0.12	Not reported	RFT = 29 (25-33), GDT = 18 (17-21)
Diaper (2020)	Single-centre RCT (Switzerland)	Major abdominal surgery	RFT versus GDTLid	Intraoperative	RFT = 2-4 mL/kg/hour balanced crystalloids, GDT = LiDCO monitor, 2-4 mL/kg/hour balanced crystalloids plus fluid boluses until increase in SVI < 10%	RFT = 201, GDT = 200	RFT = 64, GDT = 65	RFT = 1, GDT = 2	RFT = 89.4%, GDT = 88.3%	RFT = 13 (8-19), GDT = 12 (8-20)
Joosten (2018)	Single-centre RCT (Belgium)	Moderate-risk abdominal surgery	RFT versus GDTClear	Intraoperative	RFT = 4 mL/kg/hour, GDT = Clearsight closed loop system, 100 mL fluid boluses, SVV < 13% and CI > 2.5 L/minute/m^2^	RFT = 19, GDT = 20	Not reported	RFT = 0, GDT = 0	Not reported	RFT = 3 (2-5), GDT = 3 (2-6)
Phan (2014)	Single-centre RCT (Australia)	Elective colorectal surgery	RFT versus GDTOD	Intraoperative	RFT = 5 mL/kg/hour LR, GDT = 5 mL/kg/hour LR, with oesophageal Doppler, fluid boluses until increase in SV < 10%	RFT = 50, GDT = 50	RFT = 65, GDT =63.1	RFT = 1, GDT = 0	Not reported	RFT = 6 (4-9), GDT = 6.5 (5-9)
Srinivasa (2013)	Single-centre RCT (New Zealand)	Elective colorectal surgery	RFT versus GDTOD	Intraoperative	RFT = 1,500 mL crystalloid, 500 mL colloid maximum, GDT = 1,500 mL crystalloid, oesophageal Doppler, fluid boluses until increase in SV < 10%	RFT = 43, GDT = 42	RFT = 72, GDT = 69	RFT = 0, GDT = 0	Not reported	RFT = 5 (2-49), GDT = 6 (3-41), P = 0.570
Zatloukal (2017)	Single-centre RCT (Czech Republic)	Liver resection surgery	RFT versus GDTFlo	Intraoperative	RFT = 1 mL/kg/hour, GDT = 2 mL/kg/hour with FloTrac, to maintain low flow state (CI < 2 L)	RFT = 17, GDT = 17	RFT = 63, GDT = 59	RFT = 0, GDT = 1, P = 0.31	Not reported	RFT = 10 (8-14), GDT = 8 (7-11)
Zhang (2012)	Single-centre RCT (China)	Major abdominal surgery	RFT versus GDTPpv	Intraoperative	RFT = 4 mL/kg/hour LR, GDT = 4 mL/kg/hour LR, fluid bolus if PPV > 11%	RFT = 20, GDT = 20	RFT = 53.3, GDT = 56.7	RFT = 0, GDT = 0	Not reported	RFT = 10.9 (+/- 1.2), GDT = 11.9 (+/- 1.2), P < 0.001
RFT versus LFT										
Barak (2006)	Single-centre RCT (Israel)	Major abdominal surgery	RFT versus LFT	Intraoperative	RFT = positive balance 0-1,000 mL, LFT = positive balance 1,000-2,000 mL	RFT = 14, LFT = 18	RFT = 59, LFT = 65	RFT = 0, LFT = 0	Not reported	RFT = 16 (11-30), LFT = 17 (8-28)
Grant (2016)	Single-centre RCT (USA)	Pancreatectomy	RFT versus LFT	Intraoperative, postoperative	RFT = 6 mL/kg/hour with 100 mL fluid bolus, postoperative 60 mL/hour, LFT = 12 mL/kg/hour with 250 mL bolus, postoperative 125 mL/hour	RFT = 166, LFT = 164	RFT = 65, LFT = 65	RFT = 1, LFT = 1* (60-day mortality)	Not reported	RFT = 7 (3-34), LFT = 7 (3-57)
Holte, Kristensen (2007)	Single-centre RCT (Denmark)	TKR (ERAS)	RFT versus LFT	Intraoperative	RFT = no preload, 10 mL/kg/hour LR, LFT = 10 mL/kg preload, 30 mL/kg/hour LR	RFT = 24, LFT = 24	RFT = 71.5, LFT = 71.5	RFT = 0, LFT = 0	Not reported	RFT = 4 (3-18), LFT = 4 (3-5)
Holte, Foss (2007)	Single-centre RCT (Denmark)	Colorectal surgery (ERAS)	RFT versus LFT	Intraoperative	RFT = no preload, 7 mL/kg/hour LR followed by 5 mL/kg/hour, LFT = preload 10 mL/kg followed by 18 mL/kg/hour	RFT = 16, LFT = 16	RFT = 73.5, LFT = 76.5	RFT = 0, LFT = 0	Not reported	RFT = 4 (2-39), LFT = 2.5 (2-9), P = 0.03
Kabon (2005)	Multicentre RCT (USA)	Colorectal surgery	RFT versus LFT	Intraoperative	RFT = 8-10 mL/kg/hour LR, LFT = 10 mL/kg preload, 16-18 mL/kg/hour	RFT = 124, LFT = 129	RFT = 53, LFT = 52	RFT = 0, LFT = 0	Not reported	RFT = 7.3 +/- 4.0, LFT = 7.0 +/- 5.4
Kalyan (2013)	Single-centre RCT (UK)	Abdominal surgery	RFT versus LFT	Intraoperative, postoperative	RFT = 1.5 mL/kg/hour, postoperative 1 mL/kg/hour, LFT = preload with 500 mL, 7 mL/kg/hour for the first hour followed by 5 mL/kg/hour, postoperative 1.5 mL/kg/hour	RFT = 119, LFT = 121	RFT = 70, LFT = 70	RFT = 2, LFT = 4	Not reported	RFT = 8 (6-11), LFT = 8 (7-12)
Kumar (2020)	Single-centre RCT (India)	Robotic colorectal surgery	RFT versus LFT	Intraoperative	RFT = 2 mL/kg/hour crystalloid, LFT = 4 mL/kg/hour crystalloid	RFT = 20, LFT = 20	RFT = 58.3, LFT = 60.3	RFT = 0, LFT = 0	Not reported	Not reported
Nisanevich (2005)	Single-centre RCT (Israel)	Abdominal surgery	RFT versus LFT	Intraoperative	RFT = 4 mL/kg/hour LR, LFT = preload with 10 mL/kg followed by 12 mL/kg/hour LR	RFT = 77, LFT = 75	RFT = 62.8, LFT = 59.4	RFT = 0, LFT = 0	Not reported	RFT = 8 (6-21), LFT = 9 (7-24), P = 0.01
Myles (2018)	Multicentre RCT (international)	Major abdominal surgery	RFT versus LFT	Intraoperative, postoperative	RFT = 5 mL/kg/hour, postoperative 0.8 mL/kg/hour, LFT = preload with 10 mL/kg, 8 mL/kg/hour, postoperative 1.5 mL/kg/hour	RFT = 1,490, LFT = 1,493	RFT = 66, LFT = 66	RFT = 31, LFT = 18 (90-day mortality), P = 0.06	RFT = 1,223 (81.9%), LFT = 1,232 (82.3%), P = 0.61	RFT = 6.4 (3.6-10.6), LFT = 5.6 (3.6-10.5), P = 0.26
Yao (2017)	Single-centre RCT (China)	Laparoscopic cholecystectomy	RFT versus LFT	Intraoperative	RFT = 5 mL/kg/hour, LFT = 30 mL/kg/hour	RFT = 50, LFT = 50	RFT = 46.04, LFT = 44.50	RFT = 0, LFT = 0	Not reported	RFT = 1, LFT = 1, P = 0.54
GDT versus SFT										
Ackland (2015)	Multicentre RCT (UK)	Major elective surgery	GDTLid versus SFT	Postoperative	GDT = LiDCO-guided, 1 mL/kg/hour LR, colloid bolus to keep SV increase < 10%, SFT = 1 mL/kg/hour with additional colloid bolus following standard parameter	GDT = 102, SFT = 102	GDT = 68, SFT = 68	GDT = 5, SFT = 5	Not reported (Kaplan-Meier curve)	Not reported
Bahlmann (2019)	Multicentre RCT (Sweden)	Transthoracic oesophageal surgery	GDTFlo versus SFT	Intraoperative	GDT = 2.5 mL/kg/hour crystalloid, FloTrac-guided colloid bolus to keep SV increase < 10%, CI > 2.5 L/minute/m^2^, SFT = no standardized protocol	GDT = 30, SFT = 29	GDT = 65, SFT = 66	GDT = 1, SFT = 1	Not reported (Kaplan-Meier curve)	GDT = 20 (15-45), SFT = 18 (15-25)
Bartha (2013)	Single-centre RCT (Sweden)	Proximal femur fracture surgery	GDTLid versus SFT	Intraoperative	GDT = 3 mL/kg/hour LR plus LiDCO-guided colloid bolus to keep SV increase < 10%, SFT = 3 mL/kg/hour LR	GDT = 74, SFT = 75	GDT = 86, SFT =85	GDT = 3, SFT = 4	Not reported	GDT = 9 (3-20), SFT = 10 (1-38)
Benes (2010)	Single-centre RCT (Czech Republic)	Elective intrabdominal surgery	GDTFlo versus SFT	Intraoperative	GDT = 8 mL/kg/hour Plasmalyte, FloTrac-guided colloid bolus to keep SV increase < 10% and dobutamine to maintain CI > 2.5 L/minute/m^2^, SFT = 8 mL/kg/hour Plasmalyte	GDT = 60, SFT= 60	GDT = 66.73, SFT = 66.32	GDT = 1, SFT = 2	Not reported	GDT = 9 (8-11.5), SFT = 10 (8-16), P = 0.0937
Bisgaard (AAA) (2013)	Single-centre RCT (Denmark)	Open abdominal aortic surgery	GDTLid versus SFT	Intraoperative	GDT = LiDCO-guided colloid bolus to keep SV increase < 10%, SFT = no standardized protocol	GDT = 32, SFT = 32	GDT = 68, SFT = 68	GDT = 1, SFT = 0	Not reported	GDT = 9 (8-11), SFT = 9 (8-14)
Bisgaard (PVA) (2013)	Single-centre RCT (Denmark)	Open lower limb vascular surgery	GDTLid versus SFT	Intraoperative	GDT = LiDCO-guided colloid bolus to keep SV increase < 10%, SFT = no standardized protocol	GDT = 20, SFT = 20	GDT = 71, SFT = 74	GDT = 0, SFT = 0	Not reported	GDT = 5.5 (3-7), SFT = 6 (3.5-9), P = 0.3
Buettner (2008)	Single-centre RCT (Germany)	Elective major abdominal surgery	GDTPic versus SFT	Intraoperative	GDT = PiCCOplus-guided fluid bolus to keep SPV < 10%, SFT = no standardized protocol	GDT = 40, SFT = 40	GDT = 61, SFT = 66	GDT = 0, SFT = 1	Not reported	GDT = 15 (6-97), SFT = 16 (6-41)
Bundgaard-Nielsen (2013)	Single-centre RCT (Denmark)	Open radical prostatectomy	GDTOD versus SFT	Intraoperative, postoperative	GDT = 50 mL/kg fluid maximum with oesophageal Doppler-guided colloid bolus to keep SV increase < 10%, postoperative 3 mL/kg/hour LR, SFT = 50 mL/kg fluid maximum	GDT = 21, SFT = 21	GDT = 63, SFT = 64	Not reported	Not reported	GDT = 3 (3-4), SFT = 3 (2-3)
Calvo-Vecino (2018)	Multicentre RCT (Spain)	Low-risk patients with major abdominal surgery	GDTOD versus SFT	Intraoperative	GDT = oesophageal Doppler-guided fluid bolus to keep SV increase < 10%, vasopressor or inotropes based on CI, SFT = 3-5 mL/kg/hour LR for laparoscopic surgery and 5-7 mL/kg/hour LR for open surgery, fluid bolus at discretion of anaesthetist	GDT = 224, SFT = 226	GDT = 66.3, SFT = 64.2	GDT = 10/209, SFT = 9/211, P = 0.81	Not reported	GDT = 5 (4-10), SFT = 7 (5-12)
Cecconi (2011)	Single-centre RCT (Italy)	Total hip arthroplasty	GDTFlo versus SFT	Intraoperative	GDT = FloTrac-guided colloid bolus to keep SV increase < 10%, SFT = preload 250 mL colloid, 10 mL/kg/hour LR	GDT = 20, SFT = 20	GDT = 69, SFT = 63	GDT = 0, SFT = 0	Not reported	GDT = 10 (9-10), SFT = 10 (9-11)
Cesur (2018)	Single-centre RCT (Turkey)	Elective colorectal surgery	GDTPVI versus SFT	Intraoperative	GDT = 2 mL/kg/hour, PVI-guided fluid bolus to keep PVI < 13%, SFT = 4-8 mL/kg/hour	GDT = 35, SFT = 35	GDT = 58.68, SFT = 62.31	Not reported	Not reported	GDT = 6 (5-7), SFT = 6 (6-7)
Challand (2012)	Single-centre RCT (UK)	Elective colorectal surgery	GDTOD versus SFT	Intraoperative	GDT = 10 mL/kg/hour LR, oesophageal Doppler-guided colloid bolus to keep SV increase < 10%, SFT = 10 mL/kg/hour LR	GDT = 89, SFT = 90	GDT = 66, SFT = 65.9	GDT = 2, SFT = 2	Not reported	GDT = 8.8 (6.0-11.9), SFT = 6.7 (4.8-13.3), P =0.09
Chytra (2007)	Single-centre RCT (Czech Republic)	Emergency trauma patients	GDTOD versus SFT	Preoperative, intraoperative, postoperative	GDT = oesophageal Doppler-guided colloid bolus to keep SV increase < 10%, blood loss replaced 1:1 colloid, SFT = 1.5 mL/kg/hour LR, blood loss replaced 1:1 colloid	GDT = 80, SFT = 82	GDT = 33, SFT = 40	GDT = 13, SFT = 18, P = 0.43	Not reported	GDT = 14 (8.25-21), SFT = 17.5 (11-29), P = 0.045
Conway (2002)	Single-centre RCT (UK)	Abdominal surgery	GDTOD versus SFT	Intraoperative	GDT = oesophageal Doppler-guided colloid bolus 3 mL/kg to keep SV increase < 10% and maintain corrected flow time > 0.35 seconds, SFT = no standardized protocol	GDT = 29, SFT = 28	GDT = 66.5, SFT = 67.5	GDT = 0, SFT = 1	Not reported	GDT = 12 (7-103), SFT = 11 (7-30)
Corbella (2018)	Single-centre RCT (Canada)	Renal transplant surgery	GDTOD versus SFT	Intraoperative	GDT = preload of 500 mL crystalloid, oesophageal Doppler-guided crystalloid bolus to keep SV increase < 10%, 0.5 mL/kg/hour, SFT = no standardized protocol	GDT = 26, SFT = 24	GDT = 58.9, SFT = 51.7	Not reported	Not reported	GDT = 14.3 (+/-13.3), SFT = 14.1 (+/-9.0), P = 0.42
Correa-Gallego (2015)	Single-centre RCT (USA)	Liver resection	R-GDTFlo versus SFT	Intraoperative	GDT = 1 mL/kg/hour crystalloid plus FloTrac-guided SVV to less than 2 standard deviations from baseline, SFT = 6 mL/kg/hour crystalloid infusion	GDT = 69, SFT = 66	GDT = 55, SFT = 58	GDT = 2, SFT = 0 (within 100 days)	Not reported	GDT = 7 (6-8), SFT = 6 (5-8), P = 0.34
Demirel (2018)	Single-centre RCT (Turkey)	Roux-en-Y gastric bypass surgery for obesity	GDTPVI versus SFT	Intraoperative	GDT = preload 500 mL crystalloid, 2 mL/kg/hour, colloid bolus to keep PVI < 14%, SFT = preload 500 mL crystalloid, 4-8 mL/kg/hour infusion	GDT = 30, SFT = 30	GDT = 36.33, SFT = 40.07	Not reported	Not reported	Not reported
Elgendy (2017)	Single-centre RCT (Egypt)	High-risk patients in major abdominal surgery	GDTFlo versus SFT	Intraoperative	GDT = FloTrac-guided colloid bolus to keep SVV < 12% and CI >2.5 L/minute/m^2^, crystalloid infusion rate not specified, SFT = infusion rate not specified, to meet conventional targets	GDT = 43, SFT = 43	GDT = 58, SFT = 57	GDT = 3, SFT = 5	Not reported	GDT = 9.7 (+/- 1.9), SFT = 12.2 (+/- 3.5), P = 0.071
El Sharkawy (2013)	Single-centre RCT (Egypt)	Liver resection	GDTOD versus SFT	Intraoperative	GDT = 6 mL/kg/hour LR infusion, oesophageal Doppler-guided colloid bolus to keep SV increase < 10%, SFT = 6 mL/kg/hour LR infusion, colloid bolus to maintain CVP, MAP and CO	GDT = 29, SFT = 30	GDT = 48.16, SFT = 50.80	GDT = 0, SFT = 0	Not reported	GDT = 6.21(+/- 0.98), SFT = 7.55 (+/- 1.82), P < 0.01
Forget (2010)	Single-centre RCT (Belgium)	Elective major abdominal surgery	GDTPVI versus SFT	Intraoperative	GDT = preload 500 mL crystalloid, 2 mL/kg/hour crystalloid infusion, colloid bolus to keep PVI < 13%, SFT = preload 500 mL crystalloid, 4-8 mL/kg/hour infusion	GDT = 41, SFT = 41	GDT = 59, SFT = 61	GDT = 2, SFT = 0	Not reported	GDT = 15.1 (14.3), SFT = 16 (17.8), P = 0.78
Funk, HayGlass (2015)	Single-centre RCT (Canada)	Open AAA surgery	GDTFlo versus SFT	Intraoperative	GDT = 3 mL/kg/hour LR infusion, FloTrac-guided colloid bolus to keep SVV < 13%, SFT = no standardized protocol	GDT = 20, SFT = 20	GDT = 70, SFT =67	GDT = 0, SFT = 2	Not reported	GDT = 8 (7-13), SFT = 8 (6-12)
Funk, Bohn (2015)	Single-centre RCT (Canada)	Free flap surgery	GDTFlo versus SFT	Intraoperative	GDT = 5 mL/kg/hour LR, FloTrac-guided colloid bolus to keep SVV < 13%, SFT = no standardized protocol	GDT = 20, SFT = 20	GDT = 47, SFT = 51	Not reported	Not reported	Not reported
Gan (2002)	Single-centre RCT (USA)	Major elective surgery	GDTOD versus SFT	Intraoperative	GDT = 5 mL/kg/hour, oesophageal Doppler-guided colloid bolus to keep SV increase < 10% and FTc > 0.35 seconds, SFT = 5 mL/kg/hour	GDT = 50, SFT = 50	GDT = 56, SFT = 59	Not reported	Not reported	GDT = 5 (+/-3), SFT = 7 (+/-3), P = 0.03
Gerent (2018)	Single-centre RCT (Brazil)	Major abdominal surgery	GDTFlo versus SFT	Postoperative	GDT = FloTrac-guided crystalloid bolus to keep CI > 2.5 L/minute/m^2^, SVI < 35 mL/m^2^, SFT = no standardized protocol	GDT = 64, SFT = 64	GDT = 66, SFT = 68	GDT = 14/64, SFT = 9/64, P = 0.4	Not reported	GDT = 11 (6-19), SFT = 10 (6-15)
Gomez-Izquierdo (2017)	Single-centre RCT (Canada)	ERAS laparoscopic colorectal surgery	GDTOD versus SFT	Intraoperative	GDT = 1.5 mL/kg/hour LR crystalloid infusion, oesophageal Doppler-guided colloid bolus to keep SV increase < 10%, SFT = 1.5 mL/kg/hour LR crystalloid infusion	GDT = 64, SFT = 64	GDT = 63, SFT = 61	GDT = 0, SFT = 0	Not reported	GDT = 4 (3-5), SFT = 4 (3-5.7), P = 0.922
Hand (2016)	Single-centre RCT (USA)	Head and neck free flap surgery	GDTFlo versus SFT	Intraoperative	GDT = weight-based crystalloid infusion, FloTrac-guided fluid bolus to keep SVV < 13 and CI > 3.0, SFT = weight-based crystalloid infusion	GDT = 47, SFT = 47	GDT = 59.1, SFT = 57.6	Not reported	Not reported	GDT = 9.11 (5.76), SFT = 10.8 (7.65), P = 0.221
Harten (2008)	Single-centre RCT (Scotland)	Emergency abdominal surgery	GDTLid versus SFT	Intraoperative	GDT = LiDCO-guided colloid bolus to keep PPV < 10%, SFT = no standardized protocol	GDT = 14, SFT = 15	GDT = 66, SFT = 64	GDT = 1/14, SFT = 2/15, P = 0.584	Not reported	GDT = 17.5 (7-41), SFT = 12 (7-55), P = 0.122
Jammer (2010)	Multicentre RCT (Norway)	Elective open colorectal surgery	GDTScv versus SFT	Intraoperative	GDT = preload 500 mL LR, then 1.5-2 mL/kg/hour LR, ScvO2-guided colloid bolus to keep ScvO2 > 75%, SFT = preload 1,000 mL LR, followed by 10-12 mL/kg/hour	GDT = 121, SFT = 120	GDT = 57, SFT = 64	GDT = 0, SFT = 0	Not reported	GDT = 13.3, SFT = 12.6
Jhanji (2010)	Single-centre RCT (UK)	Elective gastrointestinal surgery	GDTOD versus SFT	Postoperative	GDT = 1 mL/kg/hour LR with oesophageal Doppler-guided colloid bolus to keep SV increase < 10%, SFT = 1 mL/kg/hour LR with colloid bolus to keep CVP rise > 2 mmHg	GDT= 45, SFT = 45	GDT = 68, SFT = 70	GDT = 5/45, SFT = 6/45	Not reported	GDT = 14 (11-26), SFT = 15 (10-26)
Kaufmann (2017)	Single-centre RCT (Germany)	Elective thoracic surgery	GDTOD versus SFT	Intraoperative	GDT = oesophageal Doppler-guided crystalloid bolus to keep SV increase < 10%, SFT = conventional haemodynamic goals	GDT = 48, SFT = 48	GDT = 65, SFT = 65	Not reported	Not reported	GDT = 9 (5-11), SFT = 11 (9-12), P = 0.005
Kim (2018)	Single-centre RCT (Korea)	Free flap reconstruction, head and neck surgery	GDTFlo versus SFT	Intraoperative	GDT = 5 mL/kg/hour crystalloid, FloTrac-guided colloid bolus to keep SVV < 12%, CI > 2.5 L/minute/m^2^, SFT = 5 mL/kg/hour, conventional goals	GDT = 31, SFT = 31	GDT = 56, SFT = 56	Not reported	Not reported	GDT = 22 (15-27), SFT = 22 (18-30)
Kumar (2016)	Single-centre RCT (India)	Major abdominal surgery	GDTFlo versus SFT	Intraoperative	GDT = FloTrac-guided fluid bolus to keep SVV < 10%, SFT = maintain CVP 10-12 mmHg	GDT = 30, SFT = 30	GDT = 55.3, SFT = 56.36	Not reported	Not reported	GDT = 9.9 (+/- 2.68), SFT = 11.96 (+/- 5.15)
Lai (2015)	Single-centre RCT (UK)	Elective major abdominal surgery	GDTLid versus SFT	Intraoperative	GDT = LiDCO-guided colloid bolus to keep SVV < 10%, SFT = conventional goals	GDT = 109, SFT = 111	GDT = 62.5, SFT = 63.4	GDT = 3/109, SFT = 2/111	Not reported	GDT = 11.8 (11.5), SFT = 9.6 (6.8)
Lilot (2018)	Single-centre RCT (France)	Elective abdominal surgery	GDTFlo versus SFT	Intraoperative	GDT = FloTrac-guided, automated closed-loop fluid bolus to keep SVV < 13%, SFT = no standardized protocol	GDT = 24, SFT = 22	GDT = 62.1, SFT = 68.5	Not reported	Not reported	GDT = 13.6 (8.7), SFT = 17 (16), P = 0.361
Lopes (2007)	Single-centre RCT (Brazil)	High-risk surgery	GDTPpv versus SFT	Intraoperative	GDT = PPV-guided colloid boluses to keep PPV < 10%, SFT = no standardized protocol	GDT = 17, SFT = 16	GDT = 63, SFT = 62	GDT = 2/17, SFT = 5/16	Not reported	GDT = 7 (6-8.25), SFT = 17 (8-20), P < 0.01
Luo (2017)	Single-centre RCT (China)	Neurosurgery	R-GDTFlo versus SFT	Intraoperative	GDT = 3 mL/kg/hour NS, FloTrac-guided colloid bolus to keep SVV < 15% and CI > 2.5, SFT = no protocol	GDT = 73, SFT = 72	GDT = 61, SFT = 62	GDT = 4/73, SFT = 9/72	Not reported	GDT = 15 (7-23), SFT = 17 (9-27), P = 0.069
Mayer (2010)	Single-centre RCT (Germany)	High-risk abdominal surgery	GDTFlo versus SFT	Intraoperative	GDT = FloTrac-guided colloid bolus to keep SVV < 12%, SFT = conventional targets to keep CVP 8-12 mmHg	GDT = 30, SFT = 30	GDT = 73, SFT = 72	GDT = 2/30, SFT = 2/30	Not reported	GDT = 15 (12-17.75), SFT = 19 (14-23.5), P = 0.006
McKenny (2013)	Single-centre RCT (Ireland)	Elective gynaecological surgery	GDTOD versus SFT	Intraoperative	GDT = oesophageal Doppler-guided colloid bolus to keep increase in SV < 10%, SFT = conventional targets	GDT = 51, SFT = 50	GDT = 58, SFT = 58	Not reported	Not reported	GDT = 6 (4-25), SFT = 7 (4-42), P = 0.5
Mikor (2015)	Single-centre RCT (Hungary)	Major abdominal surgery	GDTScv versus SFT	Intraoperative	GDT = ScvO2-guided colloid bolus to keep ScvO2 > 75%, SFT = conventional targets	GDT = 38, SFT = 41	GDT = 62, SFT = 62	GDT = 1/39, SFT = 8/41, P = 0.018	Not reported	Not reported
Moppett (2015)	Single-centre RCT (UK)	Hip fracture	GDTLid versus SFT	Intraoperative	GDT = LiDCO-guided colloid bolus to keep SV increase < 10%, SFT = no standardized protocol	GDT = 51, SFT = 63	GDT = 85, SFT = 85	GDT = 0/51, SFT = 7/63	Not reported	GDT = 15.3 (13.8-17.2), SFT = 14.2 (12.9-15.8)
Noblett (2006)	Single-centre RCT (UK)	Elective colorectal resection	GDTOD versus SFT	Intraoperative	GDT = oesophageal Doppler-guided colloid bolus to keep SV increase < 10% and FTc > 350 millisecond, SFT = no standardized protocol	GDT = 51, SFT = 52	GDT = 62.3, SFT = 67.6	GDT = 0/51, SFT = 1/52	Not reported	GDT = 7 (3-35), SFT = 9 (4-45), P = 0.005
Pavlovic (2016)	Single-centre RCT (Switzerland)	Emergency surgery	GDTPic versus SFT	Intraoperative	GDT = 8-10 mL/kg NS preload, 3-4 mL/kg/hour crystalloid, PiCCOplus-guided colloid bolus to keep SVV < 12% or PPV < 12%, SFT = 8-10 mL/kg NS preload, 3-4 mL/kg/hour crystalloid	GDT = 20, SFT = 23	GDT = 63, SFT = 66	GDT = 5/20, SFT = 3/23, P = 0.540	Not reported	GDT = 31 (17-42), SFT = 27 (13-43), P = 0.955
Pearse (2014)	Multicentre RCT (UK)	Major gastrointestinal surgery	GDTLid versus SFT	Intraoperative, postoperative	GDT = LiDCO-guided colloid bolus to maintain maximal stroke volume, SFT = conventional targets, no standardized protocol	GDT = 368, SFT = 365	GDT = 71.3, SFT = 72.2	GDT = 12/366, SFT = 11/364	Not reported	GDT = 10 (7-14), SFT = 11 (7-17), P = 0.05
Pearse (2005)	Single-centre RCT (UK)	Major surgery	GDTLid versus SFT	Postoperative	GDT = 1.5 mL/kg/hour crystalloid, LiDCO-guided colloid bolus to maintain SV increase < 10% and DO2I > 600 mL/minute/m^2^, SFT = 1.5 mL/kg/hour crystalloid, maintain CVP rise of 2 mmHg	GDT = 62, SFT = 60	GDT = 66, SFT = 68	GDT = 6/62, SFT = 7/60	Not reported	GDT = 17.5 (+/- 20.8), SFT = 29.5 (+/-34.8), P = 0.001
Peng (2014)	Single-centre RCT (China)	Major orthopaedic surgery	GDTFlo versus SFT	Intraoperative	GDT = 5 mL/kg/hour LR, FloTrac-guided colloid bolus to maintain SVV < 10%, SFT = 5 mL/kg/hour LR, conventional targets	GDT = 40, SFT = 40	GDT = 55, SFT = 53	GDT = 1/40, SFT = 0/40	Not reported	GDT = 12 (+/-3), SFT = 11 (+/-7)
Pestaña (2014)	Multicentre RCT (international)	Major abdominal surgery	GDTNic versus SFT	Intraoperative	GDT = NICOM-guided colloid bolus to maintain MAP > 65 mmHg and CI > 2.5L/minute/m^2^, SFT = no standardized protocol, conventional targets	GDT = 72, SFT = 70	GDT = 73.5, SFT = 74	GDT = 3/72, SFT = 4/70	Not reported	GDT = 11.5 (8-15), SFT = 10.5 (8-16), P = 0.874
Pillai (2011)	Single-centre RCT (UK)	Radical cystectomy	GDTOD versus SFT	Intraoperative	GDT = oesophageal Doppler-guided colloid bolus to keep SVV < 10% and FTc > 350 millisecond, SFT = no standardized protocol or targets	GDT = 32, SFT = 34	GDT = 66.4, SFT = 68.5	Not reported	Not reported	GDT = 18 (14.3-21.7), SFT = 22 (18.4-25.6), P = 0.12
Ramsingh (2013)	Single-centre RCT (USA)	High-risk abdominal surgery	GDTFlo versus SFT	Intraoperative	GDT = FloTrac-guided albumin bolus to keep SVV < 12%, SFT = no standardized protocol	GDT = 18, SFT = 20	GDT = 53.5, SFT = 64.4	Not reported	Not reported	GDT = 5.0 (3.75-8.25), SFT = 7.5 (5.25-10.75), P = 0.04
Reisinger (2017)	Single-centre RCT (Netherlands)	Colorectal surgery	GDTOD versus SFT	Intraoperative, postoperative	GDT = oesophageal Doppler-guided colloid bolus to keep increase in SVI < 10%, SFT = no standardized protocol	GDT = 27, SFT = 31	GDT = 68.6, SFT = 67.6	GDT = 0/27, SFT = 1/31	Not reported	GDT = 11 (4-50), SFT = 8 (5-26)
Salzwedel (2013)	Multicentre RCT (international)	Major abdominal surgery	GDTPro versus SFT	Intraoperative	GDT = ProAQT-guided colloid bolus to keep PPV < 10% and CI > 2.5 L/minute/m^2^, SFT = no standardized protocol	GDT = 79, SFT = 81	GDT = 63, SFT = 65	Not reported	Not reported	GDT = 11 (8), SFT = 10 (11.8), P = 0.929
Scheeren (2013)	Multicentre RCT (Germany)	High-risk surgical patients	GDTFlo versus SFT	Intraoperative	GDT = FloTrac-guided colloid bolus to keep SVV < 10%, SFT = conventional targets, standardized protocol	GDT = 26, SFT = 26	GDT = 68, SFT = 73	GDT = 0/26, SFT = 2/26	Not reported	Not reported
Schmid (2014)	Single-centre RCT (Germany)	Major abdominal surgery	GDTPic versus SFT	Intraoperative, postoperative	GDT = 100 mL/hour LR, PiCCO2-guided fluid bolus to maintain GEDI > 800 and CI > 2.5, SFT = conventional targets, standardized protocol	GDT = 92, SFT = 88	GDT = 67, SFT = 65	GDT = 4/92, SFT = 4/88, P = 0.65	GDT = 55/77, SFT = 61/771	Not reported
Senagore (2009)	Single-centre RCT (USA)	Laparoscopic colorectal surgery (ERAS)	GDTOD versus SFT	Intraoperative	GDT = preload 5 mL/kg LR, 5 mL/kg/hour LR, oesophageal Doppler-guided colloid bolus to keep SVV < 10%, SFT = preload 5 mL/kg LR, 5 mL/kg/hour LR	GDT = 42, SFT = 22	Not reported	GDT = 1/42, SFT = 0/22	Not reported	GDT = 3.14, SFT = 2.7
Sinclair (1997)	Single-centre RCT (UK)	Proximal femoral fracture surgery	GDTOD versus SFT	Intraoperative	GDT = oesophageal Doppler-guided fluid bolus to keep FTc > 0.35 seconds and SVV < 10%, SFT = no standardized protocol	GDT = 20, SFT = 20	GDT = 74, SFT = 75.5	GDT = 1/20, SFT = 2/20	Not reported	GDT = 12 (8-13), SFT = 20 (10-61), P < 0.05
Stens (2017)	Multicentre RCT (Netherlands)	Moderate-risk abdominal surgery	GDTNex versus SFT	Intraoperative	GDT = ccNexfin-guided fluid bolus to keep PPV < 12% and CI > 2.5, MAP > 70 mmHg, SFT = keep MAP > 70 mmHg, no fluid protocol	GDT = 81, SFT= 94	GDT = 61, SFT = 65	GDT = 1/81, SFT = 1/94	Not reported	GDT = 6 (1-44), SFT = 6 (1-30)
Szakmany (2005)	Single-centre RCT (Hungary)	Major abdominal surgery	GDTITBV versus SFT	Intraoperative	GDT = 10 mL/kg/hour LR, ITBV-guided colloid bolus to keep ITBV index 850-950 mL/m^2^, SFT = 10 mL/kg/hour LR, CVP-guided colloid bolus to keep CVP 8-12 mmHg	GDT = 20, SFT = 20	GDT = 61, SFT = 59	GDT = 2/20, SFT = 1/20	Not reported	Not reported
Szturz (2018)	Single-centre RCT (Czech Republic)	Intermediate-risk open gastrointestinal surgery	GDTOD versus SFT	Intraoperative	GDT = oesophageal Doppler-guided crystalloid bolus to keep FTc > 330 millisecond, CI > 2.5, SFT = standardized protocol, conventional targets	GDT = 71, SFT = 60	GDT = 66, SFT = 65	GDT = 1/71, SFT = 1/71	Not reported	GDT = 9 (6-21), SFT = 11 (6-40), P = 0.301
Van der Linden (2010)	Single-centre RCT (Belgium)	Peripheral arterial surgery	GDTFlo versus SFT	Intraoperative	GDT = FloTrac-guided colloid bolus to keep CI > 2.5 L/m^2^/minute, SFT = conventional targets, no fluid protocol	GDT = 40, SFT = 17	GDT = 76, SFT = 68	GDT = 3/40, SFT = 0/17	Not reported	GDT = 18 (12-26), SFT = 14 (13-19)
Venn (2002)	Single-centre RCT (UK)	Hip fracture surgery	GDTOD versus SFT	Intraoperative	GDT = oesophageal Doppler-guided colloid bolus to maintain SVV < 10% and FTc > 0.4 seconds, SFT = no fluid protocol, standard targets	GDT = 30, SFT = 29	GDT = 82, SFT = 84.5	GDT = 3/30, SFT = 2/30	Not reported	GDT = 13.5 (10.9-17.5), SFT = 17.5 (13.9-24.4)
Wakeling (2005)	Single-centre RCT (UK)	Major bowel surgery	GDTOD versus SFT	Intraoperative	GDT = oesophageal Doppler-guided colloid bolus to keep SV increase < 10% and CVP rise > 3 mmHg, SFT = conventional targets, no standardized protocol	GDT = 64, SFT = 64	GDT = 69.1, SFT = 69.6	GDT = 0/64, SFT = 0/64	Not reported	GDT = 10 (+/-5.75), SFT = 11.5 (+/-4.75), P = 0.031
Weinberg (2017)	Single-centre RCT (Australia)	Pancreaticoduodenectomy	R-GDTFlo versus SFT	Intraoperative	GDT = FloTrac-guided crystalloid bolus to keep SVV < 20%, CI > 2 L/minute/m^2^, SFT = no standardized protocol	GDT = 26, SFT = 26	GDT = 61, SFT = 68	GDT = 0/26, SFT = 0/26	Not reported	GDT = 9.5 (7.0-14.3), SFT = 12.5 (9-22.3), P = 0.002
Weinberg (2019)	Multicentre RCT (Australia)	Major liver resection	R-GDTFlo versus SFT	Intraoperative	GDT = FloTrac-guided crystalloid bolus to keep SVV < 20% during liver transection, <15% during haemostasis and closure, SFT = low CVP < 8 mmHg, no standardized protocol	GDT = 24, SFT = 24	GDT = 64, SFT = 61	GDT = 0/24, SFT = 0/24	Not reported	GDT = 7 (6-8), SFT = 8 (6-10), P = 0.17
Wu (2017)	Single-centre RCT (China)	Intracranial tumour surgery	R-GDTFlo versus SFT	Intraoperative	GDT = 3 mL/kg colloid preload, 4+2+1 mL/kg/hour crystalloid, FloTrac-guided colloid bolus to keep SVV < 12% and CI > 2.5 L/minute/m^2^, SFT = 3 mL/kg colloid preload, 4+2+1 mL/kg/hour crystalloid, keep CVP > 8 mmHg and conventional targets	GDT = 33, SFT = 30	GDT = 50, SFT = 50	Not reported	Not reported	GDT = 10.4 (3.9), SFT = 12.2 (5.1)
Xu (2017)	Single-centre RCT (China)	Thoracic surgery with one-lung ventilation	R-GDTFlo versus SFT	Intraoperative	GDT = 4 mL/kg/hour crystalloid infusion, FloTrac-guided colloid bolus to keep SVV < 13% and CI > 2.5 L/minute/m^2^, SFT = 4 mL/kg/hour crystalloid infusion, conventional targets	GDT = 84, SFT = 84	GDT = 49, SFT = 49	Not reported	Not reported	GDT = 7 (6-8), SFT = 8 (7-9), P < 0.001
Zakhaleva (2013)	Single-centre RCT (USA)	Bowel surgery (ERAS)	GDTOD versus SFT	Intraoperative	GDT = 4-8 mL/kg/hour crystalloid infusion, oesophageal Doppler colloid bolus to keep FTc > 350 millisecond and SV increase < 10%, SFT = 4-8 mL/kg/hour crystalloid infusion	GDT = 32, SFT = 40	GDT = 57, SFT = 57	GDT = 0, SFT = 0	Not reported	GDT = 6 (3-30), SFT = 5 (3-16), P = 0.575
Zhang (2013)	Single-centre RCT (China)	Thoracic surgery with one-lung ventilation	GDTFlo versus SFT	Intraoperative	GDT = 8 mL/kg/hour crystalloid infusion, FloTrac-guided colloid bolus to keep SVV < 11% and CI > 2.5 L, SFT = 8 mL/kg/hour crystalloid infusion, conventional targets	GDT = 30, SFT = 30	GDT = 59.9, SFT = 61	Not reported	Not reported	GDT = 4.5, SFT = 5
Zhao (2018)	Single-centre RCT (China)	Gastrointestinal surgery	R-GDTFlo versus SFT	Intraoperative	GDT = 3 mL/kg/hour crystalloid infusion, FloTrac-guided colloid bolus to keep SVV < 13%, SFT = conventional targets, no standardized protocol	GDT = 44, SFT = 44	GDT = 67.77, SFT = 70.41	Not reported	Not reported	GDT = 21.93 (9.34), SFT = 26.16 (7.12)
Zheng (2013)	Single-centre RCT (China)	Gastrointestinal surgery	GDTFlo versus SFT	Intraoperative	GDT = 5-7 mL/kg crystalloid preload, FloTrac-guided crystalloid bolus to keep SVV < 12%, CI > 2.5 L/minute/m^2^, SFT = 5-7 mL/kg crystalloid preload, 4-2-1 mL/kg/hour crystalloid infusion	GDT = 30, SFT = 30	GDT = 68, SFT = 67	GDT = 0, SFT = 0	Not reported	GDT = 18 (16-22.25), SFT = 22 (19-27)

The risks of bias within the individual studies are presented in Table 3.

**Table 3:** Risks of bias within individual studies Low (+), low risk of bias; high (-), high risk of bias; unclear (?), unclear risk of bias according to the relative information RFT, restricted fluid therapy; SFT, standard fluid therapy; GDT, goal-directed therapy; LFT, liberal fluid therapy

Study	Selection bias		Performance bias	Detection bias	Attrition bias	Reporting bias	Other bias	Overall bias
Author (year)	Random sequence generation	Allocation concealment	Blinding of participants and personnel	Blinding of outcome assessment	Incomplete outcome data	Selective reporting		
RFT versus SFT								
Abraham-Nordling (2012)	+	+	+	+	+	+	+	+
Brandstrup (2003)	+	+	+	+	+	+	+	+
Cohn (2010)	+	+	?	+	+	+	?	?
Futier (2010)	+	+	+	+	+	+	+	+
Gao (2012)	+	-	?	-	?	+	?	-
González-Fajardo (2009)	+	?	-	+	+	+	?	?
Jie (2014)	+	+	-	-	?	?	-	-
Lavu (2014)	+	+	+	+	+	+	+	+
Lobo (2011)	+	+	+	?	+	+	+	?
MacKay (2006)	+	+	-	+	+	+	+	+
McArdle (2009)	+	+	-	+	+	+	?	?
Piljic (2016)	+	?	-	-	?	?	?	-
Van Samkar (2015)	+	+	+	+	+	+	+	+
Vermeulen (2009)	+	+	+	+	+	+	+	+
Wuethrich (2014)	+	+	+	+	+	+	+	+
RFT versus GDT								
Benes (2015)	+	+	?	+	+	+	+	+
Brandstrup (2012)	+	+	+	+	+	+	+	+
Colantonio (2015)	+	?	-	+	?	?	?	-
Joosten (2018)	+	+	+	+	+	+	?	+
Phan (2014)	+	+	?	+	+	+	+	+
Srinivasa (2013)	+	+	+	+	+	+	+	+
Zatloukal (2017)	+	+	?	+	?	?	?	-
Zhang (2012)	+	+	-	-	-	?	?	-
RFT versus LFT								
Barak (2006)	+	-	-	-	?	?	?	-
Grant (2016)	+	?	-	-	?	?	-	-
Holte, Kristensen (2007)	+	+	+	+	+	+	+	+
Holte, Foss (2007)	+	+	+	+	+	+	+	+
Kabon (2005)	+	+	+	+	?	+	+	+
Kalyan (2013)	+	+	+	+	+	+	+	+
Kumar (2020)	+	+	+	?	-	?	?	-
Nisanevich (2005)	+	?	?	+	+	+	+	+
Myles (2018)	+	+	-	+	+	+	?	+
Yao (2017)	+	+	?	?	?	?	?	-
GDT versus SFT								
Ackland (2015)	+	+	+	+	+	+	+	+
Bahlmann (2019)	+	+	+	+	+	+	+	+
Bartha (2013)	+	+	-	-	+	+	?	?
Benes (2010)	+	+	-	+	+	+	+	?
Berlauk (1991)	+	?	-	-	+	?	?	-
Bisgaard AAA (2013)	+	+	?	+	+	+	?	?
Bisgaard PVA (2013)	+	+	?	+	+	+	?	?
Buettner (2008)	+	+	?	+	+	+	?	+
Bundgaard-Nielsen (2013)	+	+	+	+	+	+	+	+
Calvo-Vecino (2018)	+	+	+	+	+	+	+	+
Cecconi (2011)	+	+	?	+	+	+	+	+
Cesur (2018)	+	+	+	-	+	+	+	?
Challand (2012)	+	+	+	+	+	+	+	+
Chytra (2007)	+	+	-	-	+	+	+	-
Conway (2002)	+	?	?	?	+	+	?	-
Corbella (2018)	+	+	+	+	+	+	+	+
Correa-Gallego (2015)	+	+	?	-	+	+	?	?
Demirel (2018)	+	?	-	-	+	+	?	-
Diaper (2020)	+	+	?	+	+	+	+	+
Elgendy (2017)	+	+	?	+	+	?	+	?
El Sharkawy (2013)	+	+	+	+	+	?	?	?
Forget (2010)	+	?	?	+	+	+	?	?
Funk, HayGlass (2015)	+	+	+	+	+	+	+	+
Funk, Bohn (2015)	+	+	+	+	+	?	?	?
Gan (2002)	+	+	-	+	+	+	+	?
Gerent (2018)	+	+	-	+	+	+	+	?
Gomez-Izquierdo (2017)	+	+	-	+	+	+	+	?
Hand (2016)	+	?	-	-	+	+	+	-
Harten (2008)	+	-	-	+	+	+	+	-
Jammer (2010)	+	+	-	+	+	+	+	?
Jhanji (2010)	+	+	?	+	+	+	+	+
Kaufmann (2017)	+	+	?	+	+	+	+	+
Kim (2018)	+	+	-	+	+	+	+	?
Kumar (2016)	+	+	?	+	+	?	?	?
Lai (2015)	+	+	+	+	+	+	+	+
Lilot (2018)	+	+	?	?	+	+	?	?
Lopes (2007)	+	+	?	-	+	?	?	-
Luo (2017)	+	+	-	+	+	+	?	?
Mayer (2010)	+	+	?	+	+	+	+	+
McKenny (2013)	+	+	?	+	+	+	+	+
Mikor (2015)	+	+	+	?	+	+	+	+
Moppett (2015)	+	+	?	+	+	+	+	+
Noblett (2006)	+	+	+	+	+	+	+	+
Pavlovic (2016)	+	+	?	+	+	+	+	+
Pearse (2005)	+	+	?	+	+	+	+	+
Peng (2014)	+	?	-	+	+	+	?	-
Pestana (2014)	+	+	?	+	+	+	+	+
Pillai (2011)	+	+	+	+	+	+	+	+
Ramsingh (2013)	+	+	?	+	+	+	+	+
Reisinger (2017)	+	-	?	+	+	+	+	?
Salzwedel (2013)	+	+	-	?	+	+	+	?
Scheeren (2013)	+	+	?	+	+	+	+	+
Schmid (2014)	+	?	?	?	+	+	+	-
Senagore (2009)	+	+	+	+	+	+	+	+
Sinclair (1997)	+	?	+	?	+	+	+	?
Stens (2017)	+	+	+	?	+	+	+	+
Szakmany (2005)	+	+	?	?	+	+	+	?
Szturz (2018)	+	+	?	+	+	+	+	+
Van der Linden (2010)	+	+	+	+	+	+	+	+
Venn (2002)	+	+	-	-	+	+	+	-
Wakeling (2005)	+	+	?	+	+	+	+	+
Weinberg (2017)	+	?	?	+	+	+	+	?
Weinberg (2019)	+	+	+	+	+	+	+	+
Wu (2017)	+	+	?	+	+	?	?	?
Xu (2017)	+	+	+	+	+	+	?	?
Zakhaleva (2013)	+	+	-	-	+	?	?	-
Zhang (2013)	+	+	-	-	+	?	?	-
Zhao (2018)	+	?	-	-	?	?	?	-
Zheng (2013)	+	?	-	+	?	?	?	-

Figure 8 presents network meta-regression forest plots that show the estimated effect (mean difference) of the interventions in comparison with SFT. It showed a minimal effect of risk of bias in all analyses.
